# Prediction of protein motions from amino acid sequence and its application to protein-protein interaction

**DOI:** 10.1186/1472-6807-10-20

**Published:** 2010-07-13

**Authors:** Shuichi Hirose, Kiyonobu Yokota, Yutaka Kuroda, Hiroshi Wako, Shigeru Endo, Satoru Kanai, Tamotsu Noguchi

**Affiliations:** 1 Computational Biology Research Center (CBRC), National Institute of Advanced Industrial Science and Technology (AIST),2-42, Aomi, Koto-ku, Tokyo, 135-0064, Japan; 2 Research Institute of IT Biology/Faculty of Science and Engineering, Waseda University,2-42, Aomi, Koto-ku, Tokyo, 135-0064, Japan; 3 Department of Biotechnology and Life Science, Graduate School of Engineering, Tokyo University of Agriculture and Technology,12-24-16, Naka-machi, Koganei-shi, Tokyo, 184-8588, Japan; 4 School of Social Sciences, Waseda University,1-6-1, Nishi-Waseda, Shinjuku-ku, Tokyo, 169-8050, Japan; 5 Department of Physics, School of Science, Kitasato University,1-15-1, Kitasato, Sagamihara, 228-8555, Japan; 6 PharmaDesign, Inc.,2-19-8, Hatchobori, Chuo-ku, Tokyo, 104-0032, Japan

## Abstract

**Background:**

Structural flexibility is an important characteristic of proteins because it is often associated with their function. The movement of a polypeptide segment in a protein can be broken down into two types of motions: internal and external ones. The former is deformation of the segment itself, but the latter involves only rotational and translational motions as a rigid body. Normal Model Analysis (NMA) can derive these two motions, but its application remains limited because it necessitates the gathering of complete structural information.

**Results:**

In this work, we present a novel method for predicting two kinds of protein motions in ordered structures. The prediction uses only information from the amino acid sequence. We prepared a dataset of the internal and external motions of segments in many proteins by application of NMA. Subsequently, we analyzed the relation between thermal motion assessed from X-ray crystallographic B-factor and internal/external motions calculated by NMA. Results show that attributes of amino acids related to the internal motion have different features from those related to the B-factors, although those related to the external motion are correlated strongly with the B-factors. Next, we developed a method to predict internal and external motions from amino acid sequences based on the Random Forest algorithm. The proposed method uses information associated with adjacent amino acid residues and secondary structures predicted from the amino acid sequence. The proposed method exhibited moderate correlation between predicted internal and external motions with those calculated by NMA. It has the highest prediction accuracy compared to a naïve model and three published predictors.

**Conclusions:**

Finally, we applied the proposed method predicting the internal motion to a set of 20 proteins that undergo large conformational change upon protein-protein interaction. Results show significant overlaps between the predicted high internal motion regions and the observed conformational change regions.

## Background

A protein molecule is not a rigid body. The scale of protein motions is very broad: motions range from local fluctuations such as those seen in loop regions to global ones involving changes in the relative position of rigid domains. Flexible regions and linkers connecting rigid regions are often observed in large proteins. Flexible regions are often necessary for proteins to perform their specific biological functions [1-4], e.g. by enabling proteins to adjust their conformations in response to external stimulation. Such stimuli can include the binding of a ligand or a change of the surrounding environment. Structural flexibility is therefore an important characteristic that must be examined to understand proteins.

When we specifically examine motions of a protein backbone segment in ordered structures, the movement is theoretically classified into two forms: internal and external motion [5]. The former is a deformation of the segment itself, but the latter involves only translational and rotational motions of the segment. In the external motion, the segment fluctuates as a rigid body by changing dihedral angles of the flanking residues. For this reason, it is considered that the internal and external motions fundamentally differ (Additional file 1: Figure S1). It is expected that the distinction between these motions will provide new insights into the relation between structural flexibility and its function [6].

Actually, NMR provides a powerful experimental technique to analyze protein dynamics at the atomic and molecular levels [7]. Particularly, NOEs and relaxation experiments provide information related to picosecond-microsecond motions of the backbone atoms [8-10]. Model-free analysis enables quantitative determination of fluctuation and slow conformational change (i.e. millisecond order) of the backbone amide vector [11,12]. The latter motion is assumed to be related to internal motion, as described above. Although NMR provides a detailed view of protein dynamics, it is time-consuming.

In contrast, computational methods are useful to calculate the dynamics of proteins for which structures are available. One method is to compare structures of a protein crystallized under different conditions or different conformers of NMR. Structural differences show a flexible region [13-15]. Another computational method is to simulate protein dynamics. Among several methods, Normal Mode Analysis (NMA) provides a straightforward means of calculating the dynamics from its structure. Although NMA is less CPU-intensive than other computer simulation methods such as Molecular Dynamics (MD), Monte-Carlo (MC) simulation, and Framework Rigidity Optimized Dynamics Algorithm (FRODA)/Floppy Inclusions and Rigid Substructure Topography (FIRST) software [16,17], it can detect concerted motions of clusters of atoms and support discussion of their motions for elucidation of their functions [18-21]. Using NMA results, Nishikawa and Go examined internal and external motions of secondary structure [5], and Ishida *et al*. studied a subtilisin-eglin c complex to explore internal and external motions of enzymes and inhibitors [6].

With the increasing number of available protein structures and the development of high-performance computers, databases of protein dynamics have been constructed. In fact, *i*GNM [22] and ProMode [23] are databases of protein motion analyzed that respectively use a Gaussian Network Model (GNM) and NMA. Another database, MolMovDB [24], presents numerous graphical representations including motions of loops, domains, and subunits. In addition, DynDom [25] provides domain, hinge axes, and hinge bending residues in proteins determined from two different conformations of the same protein.

Recently, web-based tools for predicting internal motion have been developed. For example, FlexOracle [26] and HingeProt [27] predict hinge regions in proteins. Furthermore, DFprot [28] predicts main-chain deformability, which corresponds fundamentally to the internal motions described above.

However, all these tools, along with NMA, offer only limited practical use because they require knowledge of the three-dimensional structure information of a protein. Recently, a few prediction techniques that address protein motions using only amino acid sequence information have been proposed. Of those, ASP [29] and the Protein Continuum Secondary Structure Predictor [30] identify conformational switches in proteins using secondary structure information. FlexPred also predicts ordered conformational change in the protein backbone using information of sequence neighbors, evolutionary conservation, and solvent accessibility [31,32]. These definitions of protein motions are similar to those of internal motion, as described above. The support vector machine-based predictor, Wiggle, predicts functionally flexible regions defined using a coarse-grained-protein dynamic modeling approach [33].

In this paper, we present a novel method for predicting internal and external motion in ordered structures. The proposed method is based on the Random Forest (RF) algorithm using information associated with the adjacent paired amino acid residues and a predicted secondary structure. The method presents the advantage of enabling prediction of protein motions using amino acid sequence information alone as the input. The proposed method exhibits moderate correlation between predicted internal and external motions with those calculated by NMA: the respective correlation coefficients are 0.525 and 0.597. To investigate the possibility that the proposed method detects flexible regions related with protein function, we applied it to 20 proteins that undergo large conformational change upon protein-protein interaction. The results revealed, in 85% of the proteins studied, overlaps between the predicted high internal motion region and observed conformational change region.

## Results and Discussion

Herein, we describe our demonstration of the relation between B-factor derived from X-ray crystallographic studies and internal/external motion. We then present the proposed algorithm and the experimental evaluation. Finally, we applied the proposed method to a set of 20 proteins that change their conformations when interacting with other molecules.

### Thermal motion and internal/external motion

**Figure 1:**
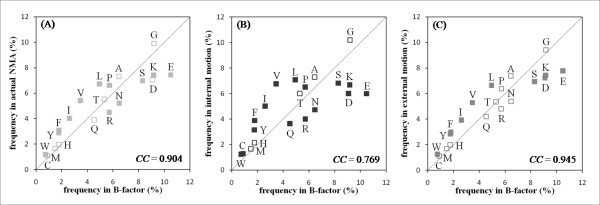
**Comparison of amino acid frequency with large fluctuations in B-factor, actual NMA value, and internal, and external motions**. (A) B-factor vs. actual NMA value, (B) B-factor vs. internal motion, and (C) B-factor vs. external motion are shown. Internal/external motion used values calculated using NMA. The horizontal and vertical axes respectively show the amino acid frequency with large fluctuations in the B-factor and actual NMA/internal/external motions. Data points close to the diagonal represent residues appearing with nearly equal frequency in B-factor and actual NMA/internal/external motions, although they deviated considerably from the diagonal represent residues appearing with different frequencies. Amino acids whose frequencies differ significantly based on *t*-test (*p *< 0.01) are shown as filled symbols. The *CC*s stand for the correlation coefficients between frequencies in B-factor and actual NMA/internal/external motions. The frequencies were estimated using 460 chains excluding NMR data from the dataset described in the Dataset section. The B-factor and three motions were normalized using the same method as that described in the Dataset section.

The B-factor determined in X-ray crystallographic studies is often used as an indicator of thermal motion. However, B-factors include both thermal motions and static deformation attributable to crystal packing and other causes. We first analyze the relation between thermal motions assessed according to the X-ray crystallographic B-factor and internal/external motions determined computationally using NMA. To this end, we calculated the correlation between the amino acid frequencies with Z-scores higher than one (i.e., residues with large motions; Figure 1). We observed that, although the overall correlation coefficients between motions as assessed using the B-factor and internal and external motions were 0.769 and 0.945, respectively, the frequencies of several amino acids differed considerably between the B-factor and both internal and external motions. Comparison of internal motion and B-factors using a *t*-test (*p *< 0.01 ) shows that the frequencies of charged amino acids E, D, K, and R are low in internal motion, whereas those of bulky or hydrophobic amino acids V, I, F, Y, and L are high (Figure 1B). Therefore, the thermal motion, as assessed using the B-factor, and internal motion have some definite discrepancies. A similar trend was apparent for the external motion and B-factor, but the difference is smaller (Figure 1C). From comparison of the actual NMA values, the external motion is shown to resemble the thermal motions.

### Length distribution of regions with a high internal/external motion score

**Figure 2:**
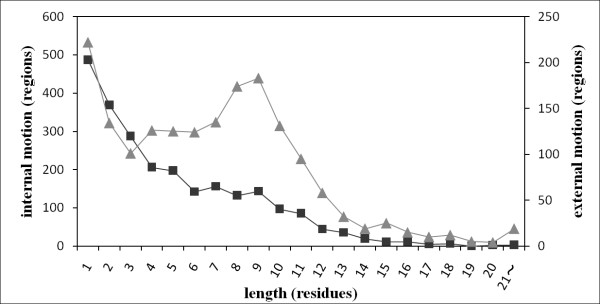
**Length distribution of consecutive amino acid with high internal/external motion scores**. Squares and triangles respectively signify internal and external motions. The length of consecutive amino acids with scores larger than one and its number are presented respectively on the horizontal and vertical axes (internal and external motions on the left and right side). These scores used the value calculated using NMA.

We investigated the length distribution of consecutive amino acids with normalized internal and external motion scores calculated using NMA larger than one (Figure 2). For both motions, the frequencies of high-mobility regions decreased as the region length increased. Results showed 3 and 19 regions longer than 21 residues long, respectively, with high internal and external motions. We noted a peak in the distribution of external motion at around nine. This observation suggests that the external motion included the short segment, which fluctuates as a rigid body such as a short helix (Additional file 1: Figure S1). According to these observations, we created two kinds of predictive models for external motion, although only one prediction model was developed for internal motion. For external motion, one model (external_short) used short flexible regions (≤ 9 residue length) as the dataset; the other model (external_long) used longer flexible regions. The final prediction result of external motion is obtained from their combination (details are presented in the Methods section).

### Construction of prediction method

We developed a prediction method for predicting internal and external motion based on Random Forest (RF) [34], which is a kind of supervised classification algorithm. The proposed method uses information about properties of the local sequence neighborhood and predicted secondary structure to predict the degrees of protein motion for a given sequence. Therefore, it is necessary to determine an optimal size of the local sequence neighborhood and suitable prediction methods that assign residues to the secondary structure (ss) and the accessible surface area (ASA). In this study, we tested two types of predictors. Amino-acid propensity based predictors constitute one type: PHD [35] and RVPnet [36] were chosen, respectively, for predicting ss and ASA. The others are profile-based predictors: psipred [37] and sable [38]. To assess the prediction accuracy of the proposed methods, three criteria were chosen: the mean absolute error (*MAE*), the correlation coefficient between prediction scores and normalized NMA scores (*CC*), and the area under the ROC curve (AUC). Their respective details are discussed in the Methods section.

**Figure 3:**
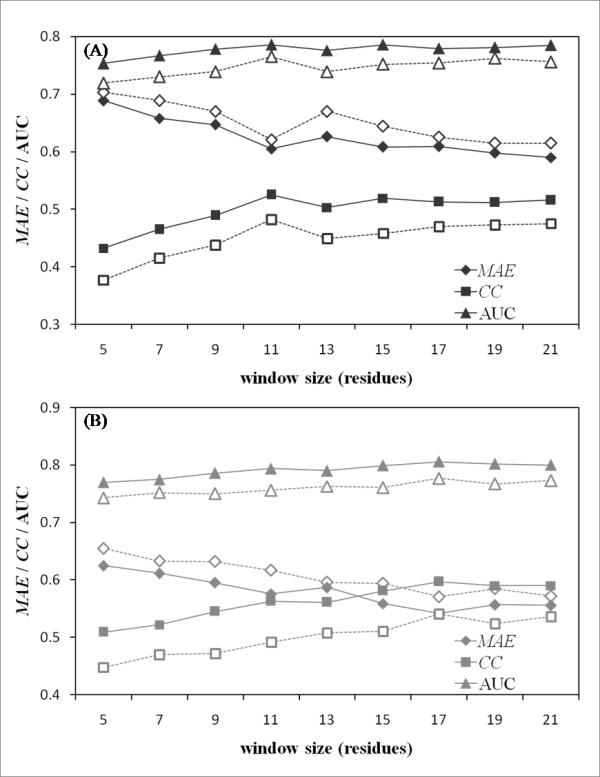
**Influence of window size and structural information predictors on prediction accuracy**. The respective performances of the proposed method for (A) internal motion and (B) external motion are shown here. The horizontal and vertical axes respectively show the window size and prediction accuracy. Filled symbols represent results obtained with the proposed method using psipred and sable; unfilled symbols signify results of the version that implemented PHD and RVPnet. The AUCs were calculated with a threshold value of 0.

We first investigated the influence of the window size and two structural information predictors on prediction accuracy (Figure 3). For internal motion, the largest gain in performance was observed when the window size was 11 residues. The optimized window sizes were the same even if structural information predictors differed. However, differences were observed between the prediction accuracies of the two methods. The prediction performance was higher when the profile-based predictors were used. In contrast, the best performance for predicting external motion was observed when the window size was 17 residues, which is larger than the window size of internal motion.

### Prediction accuracy

We compared the prediction score with normalized NMA score to assess the predictive performance. In general, protein motion for each residue tends to be large in a coil or loop region and small in a secondary structure. Based on this concept, we created a naïve model, which predicts the score that reflects the degrees of protein motions. In the naïve models, first, the secondary structure was predicted using PHD or psipred. Then, the magnitude of protein motion for each residue was calculated as follows. If the *i*-th residue is located in the secondary structure (*ss*), then

$$N_{\text{s}core}=-1.5\;\;\times \;\;SD\;\;\times \;\;\frac{1}{\left(1+\text{separation}\;\text{from}\;\text{center}\;\text{of}\;ss\right)}\;+avg.,$$

else,

$$N_{\text{s}core}=3\;\;\times \;\;SD\;\;\times \;\frac{1}{\left(1+\text{separation}\;\text{from}\;\text{center}\;\text{of}\;\text{other}\;\text{region}\right)}+avg.,$$

where *avg*. and *SD *respectively signify the average and standard deviation of the normalized NMA score in the whole dataset. Actually, 6.80e-6 and 7.49e-3 were used, respectively for *avg*. of internal and external motion; 1.00 and 1.01 were used for *SD*, respectively. The other region has no secondary structures.

We also performed comparison with three published methods that predict a region with protein motion, although their definitions of protein motion differed. First, we chose the B-factor predictor because the amino acid frequencies of the external motion and the B-factor are similar (Figure 1). In this case, we selected PROFbval, which predicts normalized backbone B-values [39]. Second, we selected disordered region, which are defined as a region lacking a stable three-dimensional structure. Although it differs from internal/external motion in terms of lacking ordered structures, they are considered to possess high flexibility. Consequently, the comparison of the proposed method with disordered region predictors is worth investigating. For predicting disordered regions, we used POODLE-S [40] in this study. Third, we specifically examined a region that is involved in a conformational switch. These regions can switch from one folded conformation to another, which is similar to the definition of internal motion in this study. To predict them, we chose FlexPred [32], which predicts residue positions that might be involved in conformational switches in ordered structures. Three predictors use only amino acid sequence information for prediction.

**Figure 4:**
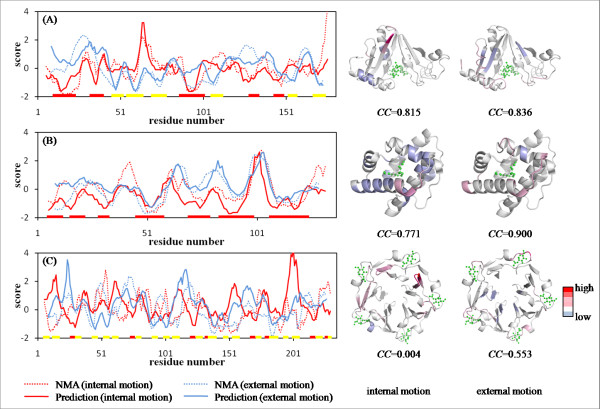
**Examples of prediction results**. Prediction results of (A) aminoglycoside 6'-N-acetyltransferase (PDB code: 1B87), (B) pheromone-binding protein (PDB code: 1DQE), and (C) tachylectin-2 (PDB code: 1TL2) are shown in the graph on the left side. The red and blue lines show predicted scores for internal and external motion; red and blue dotted lines respectively signify calculated scores obtained using NMA. The residue number is shown on the horizontal axis. The locations of secondary structures are shown as red and yellow bars, respectively, for *α*-helix and *β*-sheet. The scores of the two motions are presented on the vertical axis. On the right side, the predicted scores for internal and external motions are mapped, respectively, with a gradient from negative (blue) to positive (red) onto their structures of the left and the right sides. Green signifies a ligand. A correlation coefficient between predicted scores and normalized NMA scores is shown as *CC*. These results were obtained using the method that implemented psipred and sable.

**Figure 5:**
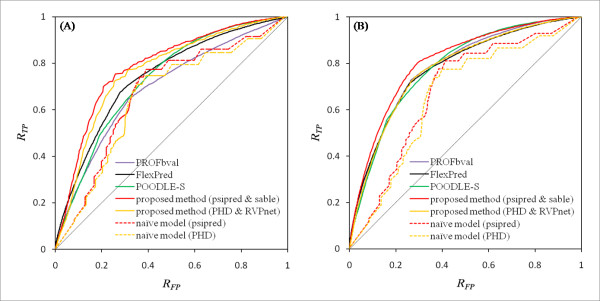
**Receiver Operator Characteristic (ROC) curves**. The ROC curves for the proposed method, naïve model, and three published predictors for (A) internal motion and (B) external motion are shown. The red and orange lines respectively signify proposed methods that implemented psipred and sable, and PHD and RVPnet. Similarly, the red and orange dashed lines respectively show the naïve model implemented with psipred and PHD. The purple, green, and black lines respectively show PROFbval, POODLE-S, and FlexPred. The threshold value was set to 0. The vertical and horizontal axes represent the true positive rate and false positive rate, as calculated in the Assessment of the Methods section.

The prediction results for three proteins are presented in Figure 4. For aminoglycoside 6'-N-acetyltransferase (Figure 4A) and pheromone-binding protein (Figure 4B), some high-mobility regions can be predicted correctly. However, some peaks were predicted incorrectly for tachylectin-2 (Figure 4C). Next, the *MAE *and *CC *of two proposed predictors were estimated by performing five-fold cross-validation tests. The proposed methods, which implemented psipred and sable, yielded the lowest scores in the averages of *MAE*s and the highest scores in the average of *CC*s among all prediction methods for both motions (Table 1). Furthermore, in three kinds of AUC that changed the threshold value, the proposed methods exhibited the best performance among them except for the threshold value of -1 for external motion (Table 1 and Figure 5). Although the naïve model assigns the same high scores equally to residues located in all loop regions, the proposed method assigns different scores to residues with dynamics.

**Table 1:** Summary of prediction accuracy for the proposed method and other methods

(A) Internal motion						
Method	*MAE*	*CC*		AUC		
	
	***avg***.	***avg***.	*SD*	1	0	-1
proposed method (PHD & RVPnet)	0.621	0.482	0.183	0.743	0.765	0.768
proposed method (psipred & sable)	0.605	0.525	0.197	0.759	0.786	0.791
naïve model (PHD)	0.988	0.248	0.161	0.633	0.653	0.688
naïve model (psipred)	0.952	0.293	0.175	0.666	0.672	0.708
PROFbval	0.743	0.367	0.199	0.711	0.693	0.698
POODLE-S	-	-	-	0.713	0.730	0.755
FlexPred	-	-	-	0.751	0.741	0.768
						

**(B) External motion**						
**Method**	***MAE***	***CC***		**AUC**		
	
	***avg*.**	***avg*.**	***SD***	**1**	**0**	**-1**

proposed method (PHD & RVPnet)	0.571	0.541	0.188	0.770	0.777	0.81
proposed method (psipred & sable)	0.542	0.597	0.209	0.806	0.806	0.843
naïve model (PHD)	0.970	0.262	0.135	0.650	0.661	0.697
naïve model (psipred)	0.929	0.320	0.145	0.685	0.681	0.733
PROFbval	0.608	0.547	0.167	0.785	0.784	0.844
POODLE-S	-	-	-	0.756	0.783	0.841
FlexPred	-	-	-	0.791	0.777	0.817

Herein, *avg*. and *SD *respectively signify the average and standard deviation. The highest scores in each criterion are underlined. Here, -1, 0, and 1 are threshold values used to discriminate rigid and flexible classes for plotting the ROC curve. PROFbval, POODLE-S, and FlexPred were performed using the default parameters. Here, POODLE-S and FlexPred respectively produced disorder probability and probability of flexible label. Therefore, *MAE *and *CC *were not calculated. The AUC for POODLE-S and FlexPred were calculated using data of normalized disorder probabilities and probabilities of flexible labels.

**Figure 6:**
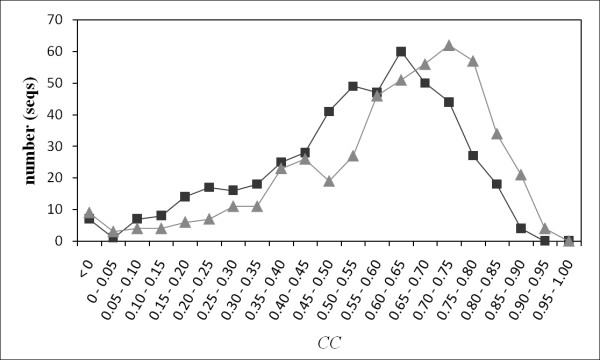
**Distribution of prediction accuracy according to the *CC***. Squares and triangles respectively represent the number of the sequences that exhibit *CC *for internal and external motions. The bin is 0.05. The *CC *and number are shown on the horizontal and vertical axes. These results were obtained using the method that implemented psipred and sable.

Therefore, the proposed method exhibited higher performance than the naïve model. Both PROFbval and POODLE-S showed higher prediction accuracy for external motion than for internal motion. In addition, the respective differences of the prediction accuracy between the proposed method for predicting external motion, and PROFbval and POODLE-S are smaller than the difference between the proposed method for predicting internal motion, and PROFbval and POODLE-S. Those results indicate that the character of the B-factor and disordered regions resembles that of external motion, as discussed above. It is noteworthy that the distribution of *CC *varied widely (Figure 6), ranging for internal and external motion from -0.185 to 0.865 and from -0.478 to 0.905, respectively. Although both *MAE *and *CC *increased roughly in relation to the margin size (Additional file 2: Figure S2), similar conclusions can be obtained for any margin.

### Relation between prediction accuracy and structure classification

**Figure 7:**
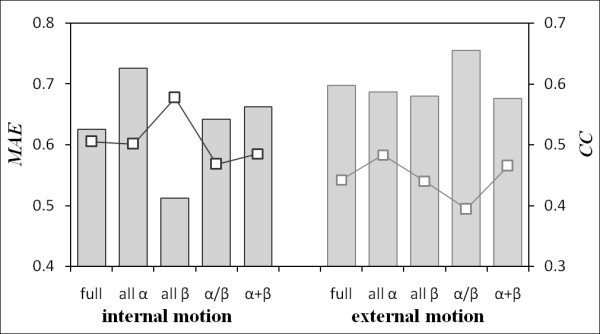
**Prediction accuracy according to SCOP classification**. Squares and bars respectively show the agerage of *MAE*s and *CC*s. Herein, full includes the full dataset; it is equal to the score shown in Table 1. The plot shows *MAE*, and the histogram shows the average of *CC*s. These results were obtained using the method that implemented psipred and sable.

In this study, we developed a novel method for predicting internal and external motion in ordered structures solely using amino acid sequence information. Although the respective performances of the proposed methods were higher than those of naïve model and three kinds of published predictors, some room exists for improving their prediction accuracies because each standard deviation of their *CC *is high (Table 1). To elucidate the cause, we investigated the prediction accuracy according to four structural groups defined by SCOP hierarchy: all-*α *protein, all-*β *protein, *α/β *protein, and *α *+ *β *protein (Figure 7). For internal motion, the all-*α *protein group exhibited the highest score in the average of *CC*s among the four groups, whereas the all-*β *protein group was the lowest. Similarly, the average of *MAE*s for all-*β *protein exhibited the highest value, which indicates poor predictive ability for its group. The prediction accuracy differed significantly among the combination of all groups and all *β *protein group, according to results of a Steel-Dwass test (*p *< 0.01). From the viewpoint of secondary structure, a similar tendency was observed: the average of *MAE*s for *β*-sheet is higher than that for *α*-helix (Additional file 3: Table S1). Therefore, the prediction result for internal motion in the proposed method was not good for tachylectin-2, which has an all-*β *structure (Figure 4C). On the other hand, no significant difference was found in the prediction accuracy for external motion among the four groups. Cases for which the proposed method incorrectly predicted high scores in *β*-sheet were often observed when predicting internal motion for all-*β *proteins. We use sliding windows with lengths of 11 and 17 residues, respectively, for predicting internal and external motion. Therefore, information about relations with two amino acids in remote positions of the sequence was ignored in this study. Consequently, it is readily conceivable that an amino acid positioned in *β*-sheet does not include sufficient information to represent its state in a three-dimensional structure in comparison with that positioned in *α*-helix because two remote amino acids of a sequence often interact in the *β*-sheet. Internal motion is deformation of the segment. Therefore, it is considered that the motion is influenced by the environment in its structure. The information of remote amino acids of a sequence is therefore necessary along with information of an adjacent amino acid of a sequence for predicting internal motion. In contrast, information of an adjacent amino acid of a sequence is thought to be more important for predicting external motion because external motion involves translational and rotational motion as a rigid body by the flanking deformed residue. Therefore, the prediction accuracy of internal motion for all-*β *proteins is thought to be low, although no difference is found in the prediction accuracy of external motion.

### Features related with protein motion

The features associated with prediction of internal and external motion in the proposed method can be evaluated according to their influence on prediction accuracy for a decreasing number of variables involved in prediction models. The RF can estimate the importance of variables more simply than commonly used machine learning methods such as SVM [34]. We investigated the relation between the prediction accuracy and the model with fewer variables using the Gini index as an indicator (Additional file 4: Figure S3).

**Figure 8:**
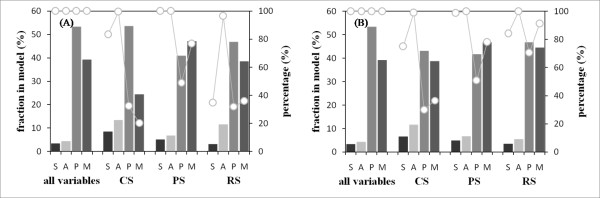
**Relation between prediction accuracy and the number of variables included in the model**. The fractions of variables included in (A) the internal motion prediction model and (B) the external_short motion prediction model. The histogram shows fractions of each variable category in the model when the variables are divided into four categories according to the feature type (see the Methods section). The plot shows percentages of the number of variables included in a downsized model against the number of variables included in the original model. The fraction of the variable category and percentage of the number of variables are respectively represented on the left and right sides' vertical axes. Although all variables were computed from the original model, the center of the secondary structure (CS), periphery of the secondary structure (PS), and remote area from the secondary structure (RS) were computed from downsized models. The CS, PS, and RS respectively signify categories which classify an amino acid according to the secondary structure (see the Methods section). Furthermore, S, A, P, and M respectively signify the names of feature groups described in the Methods section, and corresponding to secondary structure, ASA, physicochem., and mobility. The value of the Gini index was set to 1.7 in (A) and 0.7 in (B). The external_long prediction model showed similar tendencies to those of the external_short prediction model (data not shown). These results were obtained using the method that implemented psipred and sable.

Then we chose and analyzed the model with the minimum number of variables among models showing almost equal prediction accuracy to that of the model with all variables, called the original model (Figure 8). From comparison with the original model, most variables relating to structural information, namely, secondary structure and ASA, remained in all models. The ratio that they occupied in their model was higher than that in the original model. When we observed features that ranked in the top 20 based on the Gini index, ASA occupied over half of features in five of six models (Additional file 5: Figure S4). On the other hand, the ratios of variables relating to amino acid properties, namely physicochemical property (physicochem.) and protein mobility propensity (mobility), were low and different, as judged using the downsized model. These results show that the structure information indicating where an amino acid is located in its structure influences CS for both motions especially. However, structural information alone is insufficient to discriminate protein motion; PS for both motions and RS for external motion require information related to amino acid properties.

These observations showed that two kinds of information--that related to secondary structure and ASA--are highly associated with two protein motions. Therefore, when a more sophisticated secondary structure and ASA predictor, such as a profile-based predictor (psipred and sable), were used in the proposed method, the prediction accuracies of these versions were slightly higher than those of versions using a amino-acid propensity based predictor (PHD and RVPnet) (Table 1).

### Application to protein-protein interaction

Although we aim to predict internal and external motion in ordered structures in this study, as one attempt to investigate the potential for the proposed method to detect protein motion associated with protein function, we compared the predicted high protein motion region with the observed large conformation change region upon protein-protein interaction. These protein motions are regarded as associated with their protein function because they are observed along with protein-protein interaction. For the experiment, we prepared a set of 20 proteins that undergo large conformation change upon association (> 2Å *C*_*α *_RMSD) created by Dobbins *et al*., with which they demonstrated the relation between normal mode fluctuations and conformational change [41] (Table 2). We compared the internal motion with observed conformational change region because it was defined as a deformation of a segment itself in this study. To begin with, we present three kinds of typical analysis results. Specifically, the observed conformation change regions are located in a binding site, hinge region, and other regions. Secondly, we discuss the overall results.

**Table 2:** List of the large conformation change proteins

Protein name	Free	Partner protein name	Complex	RMSD (Å) (overall)	Conformational change region	RMSD (Å) (local)
Staphylococcus A	1BDD_A	Human Fc fragment	1FC2_C	3.07	41-45	3.05

Ran GTPase	1QG4_A	RCC1	1I2M_A	2.62	73-75, 126-130, 137-140	3.74

14-3-3	1QJB_AB	Serotonin N-acetylase	1IB1_AB	3.34	129-140, 201-212	8.65

Actin	1IJJ_B	Profilin	2BTF_A	2.71	37-54	5.68

Erythropoietin	1BUY_A	EPO receptor	1EER_A	4.08	112-130	5.46

Fab fragment	1GIG_LH	Flu virus hemagglutinin	2VIS_AB	4.97	105-114	3.85
						
					96-104, 119-124	

TGF-beta	1TGK_A	TGF-beta receptor	1KTZ_A	2.19	47-76	3.12

Actin	1IJJ_B	Dnase I	1ATN_A	2.71	37-54	6.14

Coagulation factor Vlla	1QFK_HL	Soluble tissue factor	1FAK_HL	6.23	153-165	5.16
						
					35-41	

Ran GTPase	1QG4_A	Importin-beta	1IBR_A	3.90	30-34, 39-42	8.56

HPr kinase C-ter domain	1JB1_A	HPr	1KKL_A	2.32	119-146	3.08

HIV1 reverse transcriptase	1S6P_AB	Fab28	2HMI_AB	3.62	69-71, 87-90, 132-134 213-224, 244-252, 291-293	3.65
						
					81-94, 217-231, 352-359	

Ecotin	1ECZ_AB	D102N trypsin	1EZU_AB	2.29	84-93	1.43

EPO receptor	1ERN_AB	Erythropoietin	1EER_BC	2.72	118-123, 125-130	3.59

Vitamin D binding	1KW2_B	Actin	1KXP_D	2.12	83-114, 247-258, 310-325	2.33

Nitrogenase Fe	2NIP_AB	Nitrogenase Mo-Fe protein	1N2C_EF	4.10	47-53, 85-88, 124-128	1.50

CDK2 kinase	1B39_A	CDK inhibitor 3	1FQ1_B	3.41	55-57, 144-165	4.33

Gelsolin	1D0N_B	Actin	1H1V_G	14.06	109-120, 210-223, 300-302	12.33

Importin-beta	1F59_A	Ran GTPase	1IBR_B	2.95	294-310, 331-338, 398-408	3.59

Hirustatin	1BX8_A	Kallikrein	1HIA_I	2.05	18-20	1.98

Proteins that undergo large conformational change upon protein-protein interaction are listed in the first column. The following information is given: PDB ID code of the free-state, partner protein name, PDB ID code of the complex-state with a partner protein, RMSD (in Å) between free-state and complex-state, observed conformational change region upon protein-protein interaction, and average RMSD (in Å) of the observed conformational change region. When a protein forms heterodimers, the observed conformational change regions were investigated in each chain.

#### (I) Ecotin

**Figure 9:**
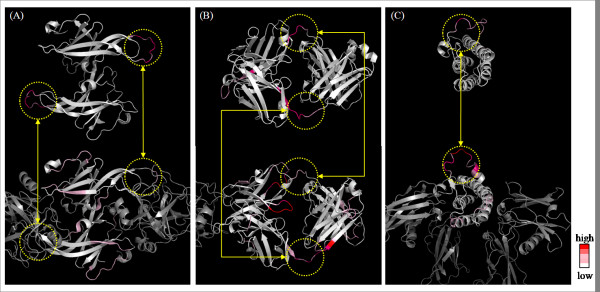
**Examples of the observed conformational change regions and predicted internal motions of (A) ecotin, (B) Fab fragment, and (C) erythropoietin**. The observed degrees of conformational change and predicted scores for internal motion are mapped, respectively, with a gradient from zero (white) to a high score (dark red) onto their structures of the upper and the lower side. The regions enclosed with a yellow dotted line correspond to the observed conformational change regions. The free-state and complex-state structures are displayed, respectively, in the upper and the lower sides. The degrees of observed conformational change were computed using the same method for calculating internal motion (see the Methods section).

Ecotin, a homodimeric protein, is an inhibitor of a group of homologous serine proteases such as trypsin, chymotrypsin, and elastase. One dimeric inhibitor binds to a protease molecule. From comparison of two structures under different crystalline environments, an inherent flexible loop was identified in the binding site with trypsin. It was necessary for its inhibitory function [42]. The proposed method predicted high internal motion on the corresponding loop (Figure 9A). FlexPred predicted a specific conformational switch region on it (Additional file 6: Figure S5A).

#### (II) Fab fragment

Fragment antigen binding (Fab fragment) is a region on an antibody that binds to antigens. It is a heterodimer of the heavy and light chains in each of the two composed domains. When Fab binds to hemagglutinin derived from a Flu virus, it was observed that the hinge region between two domains changed their conformation. That hinge movement agreed with DynDom definition, which determines the dynamic domain and hinge axes from two protein structures [25]. The proposed method predicted high internal motion at the hinge region in each chain (Figure 9B). PROFbval predicted high B-factor on the terminal of hinge region in L chain (Additional file 6: Figure S5B).

#### (III) Erythropoietin

Erythropoietin (EPO) is a hormone produced primarily in the kidneys. It has four-helical bundle topology with two long loops; it is bound to the extracellular domain of the EPO receptor. The CD loop located in the region remote from the binding site changed its conformation [43]. Additionally, ^15^N NMR relaxation data revealed a region (Leu112-Thr132) that has intrinsic flexibility [43]. The proposed method predicted high internal motion on the corresponding loop (Figure 9C). POODLE-S predicted its loop to be disordered region (Additional file 6: Figure S5C).

##### Overall results

From results obtained by application of the proposed method to a set of 20 proteins, three or more consecutive residues with predicted score higher than one were extracted. Then, they were regarded as candidates for the conformational change regions. A comparison between the observed conformational change region with predicted high internal motion region revealed at least one overlap between them in 85% of the proteins studied (Table 3, Additional file 7: Table S2 and Additional file 8: Figure S6). If the analysis object was limited to the 16 proteins that undergo conformational change for only one partner, then overlap was observed in 15 proteins (94% of the proteins studied), excepting hirustatin. The internal motion was not predicted for hirustatin because it is a small protein (55 residues). For FlexPred, which predicts conformational switches in proteins, overlaps were observed in only six proteins. Although the definition of the internal motion in this study and conformational switches in FlexPred are similar, the proposed method can detect more observed conformational change regions upon protein-protein interaction than FlexPred can. The proposed method covers all positive results of FlexPred. Both PROFbval and POODLE-S respectively predicted high scores on conformational change regions of 10 and 9 proteins. These observations suggest that the proposed method is sensitive for detection of protein motions related to protein-protein interaction, especially proteins that change conformation for a specific target.

**Table 3:** Summary of evaluation

Protein name	Partner protein name	PROFbval^1^	POODLE-S^2^	FlexPred^1^	Proposed method
Staphylococcus A	Human Fc fragment	×	○	×	○

Ran GTPase	RCC1	×	○	×	○

14-3-3	Serotonin N-acteylase	○	○	×	○

Actin	Profilin	×	×	×	×

Erythropoietin	EPO receptor	×	○	○	○

Fab fragment	Flu virus hemagglutinin	○	×	×	○

TGF-beta	TGF-beta receptor	×	○	○	○

Actin	Dnase I	×	×	×	×

Coagulation factor Vlla	Soluble tissue factor	○	×	×	○

Ran GTPase	Importin-beta	×	×	×	○

HPr kinase C-ter domain	HPr	○	○	○	○

HIV1 reverse transcriptase	Fab28	○	○	×	○

Ecotin	D102N trypsin	○	○	○	○

EPO receptor	Erythropoietin	×	×	×	○

Vitamin D bindings	Actin	○	×	○	○

Nitrogenase Fe	Nitrogenase Mo-Fe protein	○	×	×	○

CDK2 kinase	CDK inhibitor 3	×	×	×	○

Gelsolin	Actin	○	○	○	○

Importin-beta	Ran GTPase	○	×	×	○

Hirustatin	Kallikrein	×	×	×	×

'○' means that the predictor detects at least one overlap between the observed conformational change region and predicted high motion region. Conversely, '×' means that there is no overlap. PROFbval, POODLE-S, and FlexPred were performed with the default parameters; then candidate conformational change regions were determined as follows.

^1 ^Three or more consecutive residues predicted to be flexible.

^2 ^Disordered region longer than three residues.

## Conclusions

We presented a novel method for predicting internal and external motions in ordered structures based on the RF algorithm using amino acid information alone. The proposed method uses two pieces of information for prediction: the adjacent paired amino acid residues and predicted secondary structure information. The method presents the advantage of prediction using only amino acid sequence information as an input. Consequently, the method is applicable to all sequences. The proposed method exhibited the possibility of detecting protein motion related with protein-protein interaction.

## Methods

### Calculation of internal and external motions

For this study, NMA was performed using FEDER/2 [23,44]. The NMA was conducted for the energy-minimized conformation using Protein Data Bank (PDB) data as a starting conformation. In an NMA, a mean-square displacement of atom *a*, $\langle D_{a}^{2}\rangle$ in the thermal fluctuations is given as the sum of contributions from individual modes

$$\langle D_{a}^{2}\rangle =\sum_{k=1}^{N}D_{ak}^{2},$$

where **D**_*ak *_is a displacement vector of the atom *a *in the *k*-th normal mode and *N *is the number of dihedral angles used as independent variables.

We consider two conformations for a segment (we considered a segment of nine residues in this study) in each normal mode. One is the minimum-energy conformation, around which the molecule is fluctuating. The position vector of atom *a *in this minimum-energy conformation is $r_{a}^{0}$. The other is an instantaneous fluctuating conformation, in which only the *k*-th normal mode is excited to the root-mean-square thermal amplitude. The position vector of the atom *a *in this distorted conformation is $r_{a}^{0}$ + **D**_*ak*_. We bring this distorted conformation to the best-fitted position with the minimum-energy conformation purely by translational and rotational motions. The displacement vector of the atom *a *by this purely translational and rotational motion is designated as $D_{ak}^{e}$; the residual one is designated as $D_{ak}^{i}$[5]. Then, **D**_*ak *_is decomposed as

$$D_{ak}=D_{ak}^{e}+D_{ak}^{i}.$$

Superscripts *e *and *i *respectively signify *external *and *internal*. The mean square deviation of the atom *a *is given as

$$\begin{array}{c} \langle D_{a}^{2}\rangle \;=\;\sum_{k}|\;D_{ak}{|}^{2}+\sum_{k}|\;D_{ak}^{i}{|}^{2}+\sum_{k}2D_{ak}^{e}\cdot D_{ak}^{i}\\ =\;\langle |D_{a}^{e}{|}^{2}\rangle +\langle |D_{a}^{i}{|}^{2}\rangle +2\langle D_{a}^{e}\cdot D_{a}^{i}\rangle . \end{array}$$

The third term on the right-hand side of this equation was usually found to be much smaller than 1% of the first two terms in our results. Therefore, the mean-square deviation of the atom *a *is decomposed approximately into external (first term) and internal (second term) ones. The magnitudes of external and internal motions of a segment, ${\langle |D_{a}^{e}{|}^{2}\rangle }^{1/2}$ and $\;{\langle |D_{a}^{i}{|}^{2}\rangle }^{1/2}$, are defined respectively as averages of $\langle |D_{a}^{e}{|}^{2}\rangle$ and $\langle |D_{a}^{i}{|}^{2}\rangle$ over constituent atoms in the segment. As described herein, we are interested in the main-chain fluctuation. For simplicity, we consider only C*α *atoms in this decomposition (meaning that we selected data for the C*α *atoms from results obtained using NMA with a full-atom model).

### Dataset

The dataset was created by selecting protein chains from ProMode [23] as described below. First, proteins with fewer than 100 residues were removed. Then proteins whose root mean square deviation (RMSD) between the energy-minimum structure and PDB structure is more than 2Å were excluded. For proteins with common SCOP id, we selected only one of them [45]. Furthermore, multi-domain proteins defined by SCOP were excluded. Next, some proteins were discarded so that maximum pairwise sequence identity was limited to 25%. The resulting dataset comprised 481 chains (87,236 residues).

We calculated internal and external motions using NMA with a full-atom model for all proteins in the dataset. Decomposed atomic fluctuations to internal and external motions calculated by NMA values, ${y^{\prime }}_{i}$, were normalized respectively to correct for the variation among the proteins in the dataset as

$$y_{i}=\frac{{y^{\prime }}_{i}-\bar{y}}{s},\;i=1\ldots ,\;M,$$

where *M *denotes the protein length, and $\bar{y}$ and *s *respectively signify the sample mean and sample standard deviation calculated for each chain. Furthermore, *yi *is designated as the normalized NMA score of internal or external motions of the *i*-th residue.

### Variables used for encoding sequences

**Figure 10:**
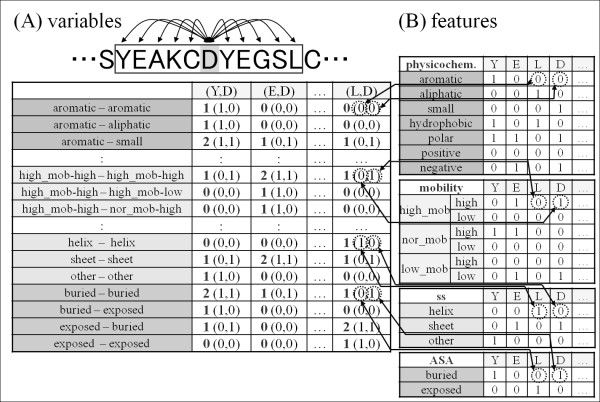
**Definitions of variables and features**. (A) Variables were obtained by adding two features shown in the first column. (L,D), (E,D), etc. respectively stand for the combinations of amino acid residues L and D, E and D, etc. The cell of the aromatic-aromatic row and the (L,D) column represents the combination of the aromatic feature of L and the aromatic feature of D shown in the physicochem. section Table (B). For instance, because the values of aromatic-L and aromatic-D are, respectively, 0 and 0, the value of the aromatic-aromatic variable for (L,D) is 0, as shown in bold. (B) Eighteen features were shown as classified into four groups: physicochem., mobility, ss, and ASA. The feature values are given for individual amino acids.

In this study, we created a prediction method that uses paired amino acid information (Figure 10). A protein sequence was encoded using a sliding window whose size is optimized for exhibiting the highest prediction performance (Figure 3; see the Results and Discussion section for details). Internal motion uses an 11-residue window. Therefore, the 920 variables (= 92 paired features × adjacent amino acids, see below for details) are defined for amino acid pairs of the central amino acid with the other 10 amino acids in the window. In contrast, the number of variables is 1,472 for external motion because the window size is 17 residues. The value of variable was given as a sum of the value of feature (defined below) of the central amino acid and paired amino acid. The value of feature was set to either 1 or 0; the value of variable can take a value of 0, 1, or 2. The five and seven residues at both termini were, respectively, excluded for internal and external motion because the value of variable is assigned to the central residue of the window.

### Features used for building variables

We defined 18 features, which were divided into four groups designated as physicochem., mobility, ss, and ASA. A value of feature of an amino acid was set to one if the amino acid satisfied a feature's definition, and to zero otherwise.

The first group was derived from physicochemical features (physicochem.) of amino acids defined according to Zvelibel *et al*. [46]. This group included seven features: hydrophobic, polar, aromatic, aliphatic, small, positive, and negative. For example, FYWH has an aromatic ring. Therefore, the value of the "aromatic" feature of Y is one (Figure 10B). For the variables, 7 × 7 = 49 pairs of these seven features are considered. The second group was derived from protein mobility propensity (mobility). This group included six features: high_mob_high, high_mob_low, nor_mob_high, nor_mob_low, low_mob_high, and low_mob_low. In determining these features, every amino acid in the dataset was classified into either the high, normal, or low mobility group defined according to normalized NMA scores which were, respectively, higher than 1, between -1 and 1, and lower than -1. Then the protein mobility propensity (*Prop*(*n,g*)) was defined as

$$Prop\;\left(n,g\right)\;=\log_{2}freq\left(n,g\right)/\sum_{g}\;freq\left(n,g\right),$$

where *freq*(*n,g*) respectively represent the relative frequencies of amino acid *n *in protein mobility group *g *(= high_mob, normal_mob, and low_mob), *g*_high = 1 or *g*_low = 1; otherwise, they are set to zero. For example, because *Prop*(*Y, normal*) is higher than one standard deviation from the average of the normal group, the value of the "nor_mob_high" feature for amino acid Y is one (Figure 10B). The meaning is that the protein mobility propensity in the normal group for Y is high. For the variables, 6 × 6 = 36 pairs of these six features are considered.

The third group was associated with the secondary structure (ss), as predicted by PHD or psipred. This group includes three features: helix, sheet, and other. If an amino acid was predicted to be in a helix region, then the value of the "helix" feature of this amino acid was one; the two other features, sheet and other, were zero (Figure 10B). For the variables, it is possible to consider 3 × 3 = 9 pairs of the three features, but we considered only three combinations in this study: helix-helix, sheet-sheet, and other-other.

The fourth group was associated with the accessible surface area (ASA) predicted using RVPnet or sable. This group includes two features: "exposed" and "buried". If an amino acid's predicted ASA value was less than 11, then the value of a "buried" feature was one; it was zero otherwise when using RVPnet. Similarly, if the predicted ASA value was higher than 27, then the value of the "exposed" feature was one (Figure 10B). Two parameters are set so that RVPnet exhibited its highest performance. When using sable, two parameters were set to one and three. For the variables, 2 × 2 = 4 pairs of the two features are considered. In summation, we consider 92 pairs of features (49, 36, 3, and 4 for four groups as described above) for each of 10 adjacent amino acid for internal motion, which results in 92 × 10 = 920 variables (Figure 10A).

### Learning method

**Figure 11:**
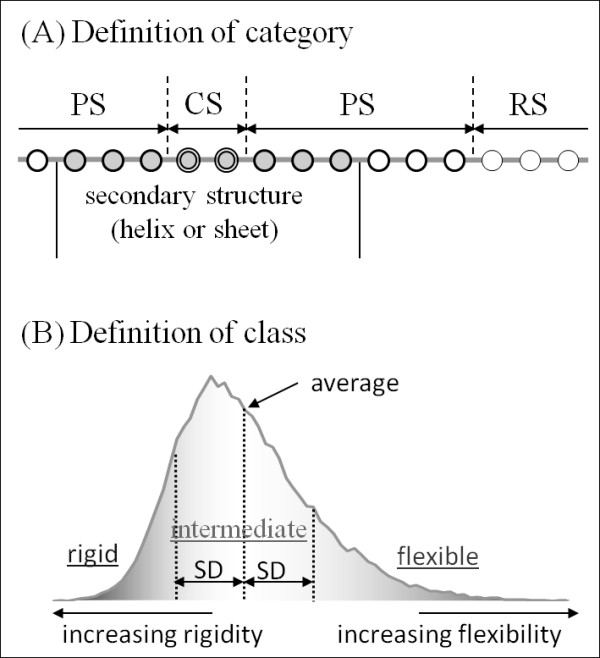
**Definition of category and class**. (A) In the definition of category, circles represent central amino acids in the windows; the filled circle shows that it appears in the secondary structure. The circles enclosed in double lines, thick lines, and thin lines respectively signify CS, PS, and RS. (B) In the definition of class, the graph represents the distribution of frequency plotted against the normalized NMA score, which is represented on the horizontal axis. The frequency is represented on the vertical axis. Average and SD respectively stand for the average value and standard deviation in each category. The graph is divided into three areas by dotted lines, indicating flexible, intermediate, and rigid classes.

Distributions of the normalized NMA score (*y_i_*) are dependent on the secondary structure type. Therefore, in this study, three kinds of models were created: one for each motion based on the idea that the degrees and tendency of mobility in proteins depend on the secondary structure. We therefore defined three categories of window locations according to the location of the central residue: center of a secondary structure (CS), remote area from secondary structures (RS), and periphery of secondary structures (PS) (Figure 11A). Hereinafter, CS means that the central residue was located in a secondary structure and three or more residues distant from either secondary structure terminus. In addition, RS signifies that the central residue was located in the other region, except for secondary structures, and three or more residues separate from both termini of the other region. Also, PS means the central amino acid was not located in either CS or RS. Furthermore, each category was divided into three classes based on the degree of the normalized NMA score: flexible, intermediate, and rigid classes (Figure 11B). A window was assigned to the intermediate class if the normalized NMA score of the central amino acid in the window was within one standard deviation from the mean. Similarly, a window was assigned to the flexible and rigid classes if the normalized NMA scores were, respectively, higher and lower than the intermediate classes.

For this study, a secondary structure was assigned to an amino acid using prediction results from either the secondary structure predictor, PHD or psipred. The amino acids predicted to form *α*-helix or *β*-sheet are considered to be located in a secondary structure. Briefly, we divided the amino acids into three categories of window locations, CS, RS, and PS. In each category, they were divided further into three classes, flexible, intermediate, and rigid.

### Prediction method

The RF algorithm was used to build a prediction model for classifying amino acids into the three classes of flexible, intermediate, and rigid. In this study, we implemented RF using the R package (randomForest 4.5-22) [47]. For internal and external motions, three RF prediction models were trained respectively for the three categories of window locations, CS, RS, and PS. Three parameters (*mtry*, *ntree*, and *nodesize*) in RF used default values because RF is robust against changes in its parameters. In fact, the prediction accuracies of the case in which the default values were used for the three parameters were almost identical to those of the case in which optimal values were used (Additional file 9: Figure S7). The results of the RF prediction model classified windows into the three classes; their prediction results were attributed to the central residue in the window. Then, the results of classification obtained from RF were converted to a score based on the average and standard deviation of the normalized NMA score in each category (Additional file 10: Table S3). For example, if a window assigned to the flexible class and the Z score of the normalized NMA score is three, then the score of the flexible class in CS of internal motion is 2.509 (= -0.248 + 0.919 × 3), and its value is given to the amino acid assigned to the flexible class in CS. The score reflects the degrees of protein motion. The score was smoothed using a five-residue moving average. Two models were used for the external motion because the length distribution of the high mobility region with NMA normalized score > 1 indicated a peak near the nine-residue-long segments (see the Results and Discussion section: Figure 2). One model (external_short) used the short flexible region (≤ 9 residue length) as the dataset of the flexible class; the other model (external_long) used a longer flexible region in the flexible class. The final prediction result of external motion combined the results of both models if the external_long model predicted a long region (> 9 residues) in the flexible class.

### Assessment

The prediction results were assessed on a residue basis: the predicted score in the sequence was compared to the normalized NMA score. The predicted scores were allowed to have a margin. In this study, we set the margin value of ± 0.2 because the minimum difference of the average of normalized NMA score between adjacent classes was 0.225 (Additional file 10: Table S3).

In this work, the error function used the mean absolute error (*MAE*), which was defined as the absolute difference between two values. Actually, *MAE *was calculated as

$$MAE=\sum |\;x_{i}-y_{i}|/M,$$

where *x*_*i *_and *y*_*i *_respectively represented the score obtained from the proposed method of *i*-th residue and the normalized NMA score; *M *denotes the protein length. The *MAE *value approaches 0 as the prediction improves.

Furthermore, to assess the method's performance, we calculated the correlation coefficient (*CC*) between two datasets as

$$CC\;\;\;=\;\;\;\frac{\sum_{i=1}^{M}\left(x_{i}-\bar{x}\right)\left(y_{i}-\bar{y}\right)}{\sqrt{\sum_{i=1}^{M}{\left(x_{i}-\bar{x}\right)}^{2}}\sqrt{\sum_{i=1}^{M}{\left(y_{i}-\bar{y}\right)}^{2}}}.$$

In fact, *CC *ranges from -1 to 1; a large positive value represents a positive correlation. In this study, it means that the patterns of the normalized NMA score and the predicted score are very similar. We also used a Receiver Operating Characteristic (ROC) curve as another assessment criterion by classifying the prediction results into flexible and rigid classes. The amino acid was assigned to the flexible class when the normalized NMA score was higher than the threshold value. The ROC curve was obtained by plotting the false positive rate (*R*_*FT*_) against the true positive rate (*R*_*TP *_). The *R*_*TP *_is defined as the percentage of windows of the flexible class correctly predicted as flexible class over all positives (sum of true positives and false positives). Similarly, the *R*_*FP *_is defined as the percentage of windows of the rigid class incorrectly predicted as flexible class over all negatives (sum of true negatives and false negatives). The *R*_*FP *_against *R*_*TP *_was shown, while the score increased from -2 to 4 with a 0.01 increment. The amino acid is considered to be predicted as a flexible class if a predicted score is higher than the score. The larger the area under the ROC curve (AUC), the more robust an algorithm is. An area of 1.00 is considered a perfect predictor.

## Authors' contributions

SH designed the prediction algorithm, performed the evaluation experiments, analyzed protein-protein interactions, and wrote the manuscript. KY provided the guidance for developing and evaluating the algorithm. YK provided a critical review and edited the manuscript. HW and SE executed NMA to calculate internal and external motion. SK provided the guidance for developing the algorithm. TN provided the guidance for evaluating experiments and analyzing protein-protein interaction. All authors contributed to the research, and mutually discussed the results and manuscript, and approved the manuscript.

## Supplementary Material

Additional file 1 **Figure S1**. Examples of internal and external motion: T7 lysozyme. Click here for file

Additional file 2 **Figure S2**. Change of the prediction accuracy according to the margin size. Click here for file

Additional file 3 **Table S1**. The average of MAEs according to the secondary structure. Click here for file

Additional file 4 **Figure S3**. Relation between the number of variables and prediction accuracy. Click here for file

Additional file 5 **Figure S4**. Proportion of features ranked in the top 20. Click here for file

Additional file 6 **Figure S5**. Prediction result of three published predictors for ection, Fab fragment, and erythropoietin. Click here for file

Additional file 7 **Table S2**. List of prediction results for the large conformational change dataset. Click here for file

Additional file 8 **Figure S6**. Distribution of internal motion and observed conformational change for 20 proteins. Click here for file

Additional file 9 **Figure S7**. Influence of three parameters of random forest on the prediction error rate. Click here for file

Additional file 10 **Table S3**. The average and standard deviation of normalized NMA score in each category. Click here for file

## References

[B1] Kirsch JF Eichele G Ford GC Vincent MG Jansonius JN Gehring H Christen P Mechanism of action of aspartate aminotransferase proposed on the basis of its spatial structure J Mol Biol 1984 174 3 497 525 10.1016/0022-2836(84)90333-4 6143829

[B2] Faber HR Matthews BW A mutant T4 lysozyme displays five different crystal conformations Nature 1990 348 6298 263 6 10.1038/348263a0 2234094

[B3] Sampson NS Knowles JR Segmental movement: definition of the structural requirements for loop closure in catalysis by triosephosphate isomerase Biochemistry 1992 31 36 8482 7 10.1021/bi00151a014 1390632

[B4] Zhang M Tanaka T Ikura M Calcium-induced conformational transition revealed by the solution structure of apo calmodulin Nat Struct Biol 1995 2 9 758 67 10.1038/nsb0995-758 7552747

[B5] Nishikawa T Go N Normal modes of vibration in bovine pancreatic trypsin inhibitor and its mechanical property Proteins 1987 2 4 308 29 10.1002/prot.340020407 3448606

[B6] Ishida H Jochi Y Kidera A Dynamic structure of subtilisin-eglin c complex studied by normal mode analysis Proteins 1998 32 3 324 33 10.1002/(SICI)1097-0134(19980815)32:3<324::AID-PROT8>3.0.CO;2-H 9715909

[B7] Williams RJ NMR studies of mobility within protein structure Eur J Biochem 1989 183 3 479 97 10.1111/j.1432-1033.1989.tb21076.x 2673776

[B8] Chi Y Kumar TK Chiu IM Yu C 15N NMR relaxation studies of free and ligand-bound human acidic fibroblast growth factor J Biol Chem 2000 275 50 39444 50 10.1074/jbc.M007205200 10982816

[B9] Chill JH Quadt SR Anglister J NMR backbone dynamics of the human type I interferon binding subunit, a representative cytokine receptor Biochemistry 2004 43 31 10127 37 10.1021/bi049606g 15287740

[B10] Gitti RK Wright NT Margolis JW Varney KM Weber DJ Margolis FL Backbone dynamics of the olfactory marker protein as studied by 15N NMR relaxation measurements Biochemistry 2005 44 28 9673 9 10.1021/bi050149t 16008352

[B11] Lipari G Szabo A Model-free approach to the interpretation of nuclear magnetic resonance relaxation in macromolecules. 1. Theory and range of validity J Am Chem Soc 1982 104 4546 59 10.1021/ja00381a009

[B12] Lipari G Szabo A Model-free approach to the interpretation of nuclear magnetic resonance relaxation in macromolecules. 2. Analysis of experimental results J Am Chem Soc 1982 104 4559 70 10.1021/ja00381a010

[B13] Shatsky M Nussinov R Wolfson HJ Flexible protein alignment and hinge detection Proteins 2002 48 2 242 56 10.1002/prot.10100 12112693

[B14] Ye Y Godzik A Database searching by flexible protein structure alignment Protein Sci 2004 13 7 1841 50 10.1110/ps.03602304 15215527 PMC2279924

[B15] Qi G Lee R Hayward S A comprehensive and non-redundant database of protein domain movements Bioinformatics 2005 21 12 2832 8 10.1093/bioinformatics/bti420 15802286

[B16] Kovacs JA Chacon P Abagyan R Predictions of protein flexibility: first-order measures Proteins 2004 56 4 661 8 10.1002/prot.20151 15281119

[B17] Wells S Menor S Hespenheide B Thorpe MF Constrained geometric simulation of diffusive motion in proteins Phys Biol 2005 2 4 S127 36 10.1088/1478-3975/2/4/S07 16280618

[B18] Go N Noguti T Nishikawa T Dynamics of a small globular protein in terms of low-frequency vibrational modes Proc Natl Acad Sci USA 1983 80 12 3696 700 10.1073/pnas.80.12.3696 6574507 PMC394117

[B19] Levitt M Sander C Stern PS Protein normal-mode dynamics: trypsin inhibitor, crambin, ribonuclease and lysozyme J Mol Biol 1985 181 3 423 47 10.1016/0022-2836(85)90230-X 2580101

[B20] Tama F Sanejouand YH Conformational change of proteins arising from normal mode calculations Protein Eng 2001 14 1 6 10.1093/protein/14.1.1 11287673

[B21] Li G Cui Q A coarse-grained normal mode approach for macromolecules: an efficient implementation and application to Ca(2+)-ATPase Biophys J 2002 83 5 2457 74 10.1016/S0006-3495(02)75257-0 12414680 PMC1302332

[B22] Yang LW Liu X Jursa CJ Holliman M Rader AJ Karimi HA Baha r I iGNM: a database of protein functional motions based on Gaussian Network Model Bioinformatics 2005 21 13 2978 87 10.1093/bioinformatics/bti469 15860562 PMC1752228

[B23] Wako H Kato M Endo S ProMode: a database of normal mode analyses on protein molecules with a full-atom model Bioinformatics 2004 20 13 2035 43 10.1093/bioinformatics/bth197 15059828

[B24] Echols N Milburn D Gerstein M MolMovDB: analysis and visualization of conformational change and structural flexibility Nucleic Acids Res 2003 31 478 82 10.1093/nar/gkg104 12520056 PMC165551

[B25] Lee RA Razaz M Hayward S The DynDom database of protein domain motions Bioinformatics 2003 19 10 1290 1 10.1093/bioinformatics/btg137 12835274

[B26] Flores SC Gerstein MB FlexOracle: predicting flexible hinges by identification of stable domains BMC Bioinformatics 2007 8 215 10.1186/1471-2105-8-215 17587456 PMC1933439

[B27] Emekli U Schneidman-Duhovny D Wolfson HJ Nussinov R Haliloglu T HingeProt: automated prediction of hinges in protein structures Proteins 2008 70 4 1219 27 10.1002/prot.21613 17847101

[B28] Garzon JI Kovacs J Abagyan R Chacon P DFprot: a webtool for predicting local chain deformability Bioinformatics 2007 23 7 901 2 10.1093/bioinformatics/btm014 17277334

[B29] Young M Kirshenbaum K Dill KA Highsmith S Predicting conformational switches in proteins Protein Sci 1999 8 9 1752 64 10.1110/ps.8.9.1752 10493576 PMC2144394

[B30] Boden M Bailey TL Identifying sequence regions undergoing conformational change via predicted continuum secondary structure Bioinformatics 2006 22 15 1809 14 10.1093/bioinformatics/btl198 16720586

[B31] Kuznetsov IB Ordered conformational change in the protein backbone: prediction of conformationally variable positions from sequence and low-resolution structural data Proteins 2008 72 74 87 10.1002/prot.21899 18186479

[B32] Kuznetsov IB McDuffie M FlexPred: a web-server for predicting residue positions involved in conformational switches in proteins Bioinformation 2008 3 3 134 6 19238251 10.6026/97320630003134 PMC2639688

[B33] Gu J Gribskov M Bourne PE Wiggle-predicting functionally flexible regions from primary sequence PLoS Comput Biol 2006 2 7 e90. 10.1371/journal.pcbi.0020090 16839194 PMC1500818

[B34] Breinman L Random Forests Machine Learning 2001 45 5 32 10.1023/A:1010933404324

[B35] Rost B PHD: predicting one-dimensional protein structure by profile-based neural networks Methods Enzymol 1996 266 525 39 full_text 8743704 10.1016/s0076-6879(96)66033-9

[B36] Ahmad S Gromiha MM Sarai A Real value prediction of solvent accessibility from amino acid sequence Proteins 2003 50 4 629 35 10.1002/prot.10328 12577269

[B37] Jones DT Protein secondary structure prediction based on position-specific scoring matrices J Mol Biol 1999 292 2 195 202 10.1006/jmbi.1999.3091 10493868

[B38] Adamczak R Porollo A Meller J Combining prediction of secondary structure and solvent accessibility in proteins Proteins 2005 59 3 467 75 10.1002/prot.20441 15768403

[B39] Schlessinger A Yachdav G Rost B PROFbval: predict flexible and rigid residues in proteins Bioinformatics 2006 22 7 891 3 10.1093/bioinformatics/btl032 16455751

[B40] Shimizu K Hirose S Noguchi T POODLE-S: web application for predicting protein disorder by using physicochemical features and reduced amino acid set of a position-specific scoring matrix Bioinformatics 2007 23 17 2337 8 10.1093/bioinformatics/btm330 17599940

[B41] Dobbins SE Lesk VI Sternberg MJ Insights into protein flexibility: The relationship between normal modes and conformational change upon protein-protein docking Proc Natl Acad Sci USA 2008 105 30 10390 5 10.1073/pnas.0802496105 18641126 PMC2475499

[B42] Shin DH Song HK Seong IS Lee CS Chung CH Suh SW Crystal structure analyses of uncomplexed ecotin in two crystal forms: implications for its function and stability Protein Sci 1996 5 11 2236 47 10.1002/pro.5560051110 8931142 PMC2143284

[B43] Cheetham JC Smith DM Aoki KH Stevenson JL Hoeffel TJ Syed RS Egrie J Harvey TS NMR structure of human erythropoietin and a comparison with its receptor bound conformation Nat Struct Biol 1998 5 10 861 6 10.1038/2302 9783743

[B44] Wako H Endo S Nagayama K Go N FEDER/2: program for static and dynamic conformational energy analysis of macro-molecules in dihedral angle space Comp Phys Comm 1995 91 233 51 10.1016/0010-4655(95)00050-P

[B45] Hubbard TJ Ailey B Brenner SE Murzin AG Chothia C SCOP, Structural Classification of Proteins database: applications to evaluation of the effectiveness of sequence alignment methods and statistics of protein structural data Acta Crystallogr D Biol Crystallogr 1998 54 (Pt 6 Pt 1):1147 54 10.1107/S0907444998009172 10089491

[B46] Zvelebil MJ Barton GJ Taylor WR Sternberg MJ Prediction of protein secondary structure and active sites using the alignment of homologous sequences J Mol Biol 1987 195 4 957 61 10.1016/0022-2836(87)90501-8 3656439

[B47] Liaw A Wiener M Classification and Regression by randomForest R News 2002 2 18 22

