# Supplementary material for: Prediction of protein motions from amino acid sequence and its application to protein-protein interaction
Source: BMC Struct Biol. 2010 Jul 13;10:20. doi: 10.1186/1472-6807-10-20 (PMC3245509; doi:10.1186/1472-6807-10-20)
Supplement: Additional file 1 — Figure S1. Examples of internal and external motion: T7 lysozyme. [file 1472-6807-10-20-S1.PDF]

Additional file 1

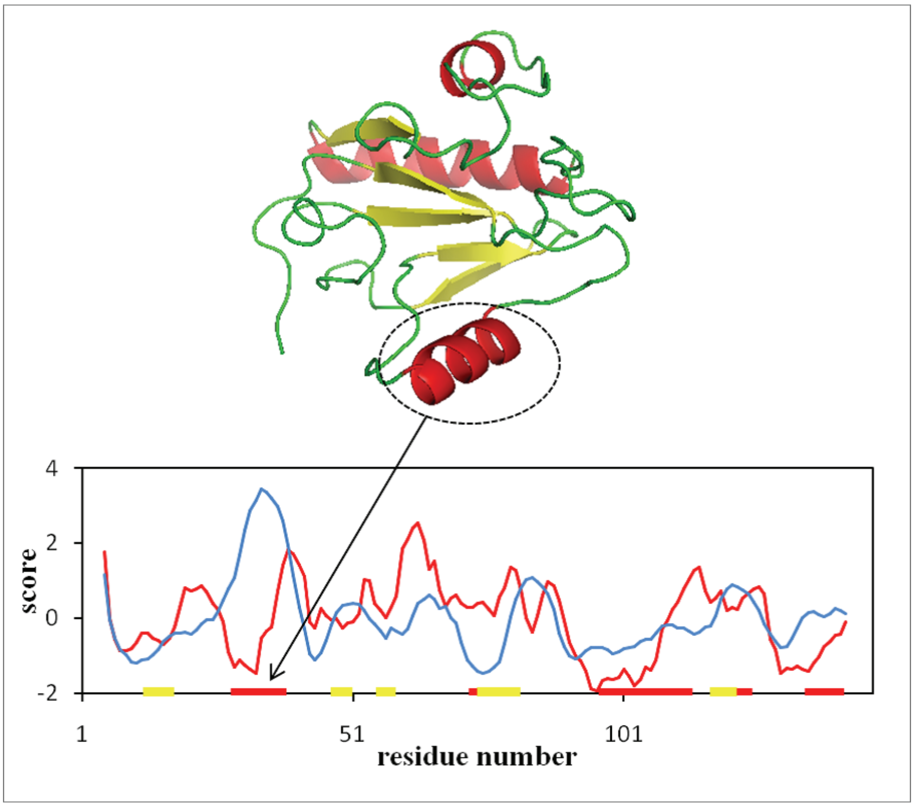

Figure S1 - Examples of internal and external motion: T7 lysozyme.

The importance of breaking down the movement of a polypeptide chain to internal and external motions is readily apparent in this example. The structure of T7 lysozyme (PDB code: 1LBA) is shown in the upper illustration; the normalized NMA scores for internal (red) and external (blue) motions are shown against residue numbers in the lower graph. The locations of secondary structures are shown as red and yellow bars, respectively, for the α-helix and β-sheet.

The comparison of the two structures under different conditions revealed that α-helix (29–38) enclosed with dotted line in the upper structure changes the relative position by translational and rotational motion. The behavior was detected by NMA, meaning that the internal motion is small, but the external motion is large in the corresponding α-helix. In this manner, it is useful to break down the movements of the segments into internal and external motions to describe the dynamic structure of proteins.
