# Supplementary material for: Prediction of protein motions from amino acid sequence and its application to protein-protein interaction
Source: BMC Struct Biol. 2010 Jul 13;10:20. doi: 10.1186/1472-6807-10-20 (PMC3245509; doi:10.1186/1472-6807-10-20)
Supplement: Additional file 2 — Figure S2. Change of the prediction accuracy according to the margin size. [file 1472-6807-10-20-S2.PDF]

Additional file 2

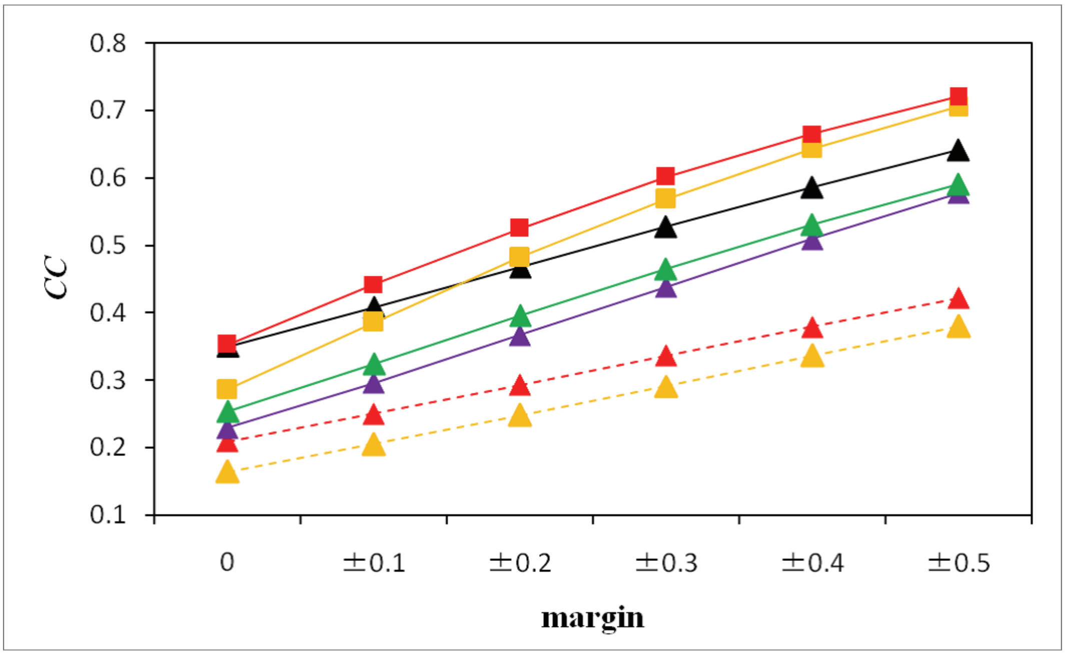

Figure S2 - Change of the prediction accuracy according to the margin size.

Red and orange squares respectively signify the average of *CC*s of the proposed method implementing psipred and sable, and PHD and RVPnet. Similarly, the red and orange triangles respectively mark the average of *CC*s of naïve model implementing psipred and PHD. The purple, green, and black triangles respectively show PROFbval, POODLE-S, and FlexPred. The margin size and average of *CC*s are shown respectively on the horizontal and vertical axes.
