# Supplementary material for: Prediction of protein motions from amino acid sequence and its application to protein-protein interaction
Source: BMC Struct Biol. 2010 Jul 13;10:20. doi: 10.1186/1472-6807-10-20 (PMC3245509; doi:10.1186/1472-6807-10-20)
Supplement: Additional file 3 — Table S1. The average of MAEs according to the secondary structure. [file 1472-6807-10-20-S3.PDF]

Additional file 3

Table S1 - The average of MAEs according to the secondary structure

(A) Internal motion

Method	Full	α helix	β sheet	Other
proposed method (PHD & RVPnet)	0.621	0.515	0.623	0.698
proposed method (psipred & sable)	0.605	0.487	0.589	0.699

(B) External motion

Method	Full	α helix	β sheet	Other
proposed method (PHD & RVPnet)	0.571	0.548	0.470	0.640
proposed method (psipred & sable)	0.542	0.520	0.444	0.608

'Other' signifies regions without a secondary structure. Herein, 'Full' comprises the full dataset; it is equal to the score presented in Table 1.
