# Supplementary material for: Prediction of protein motions from amino acid sequence and its application to protein-protein interaction
Source: BMC Struct Biol. 2010 Jul 13;10:20. doi: 10.1186/1472-6807-10-20 (PMC3245509; doi:10.1186/1472-6807-10-20)
Supplement: Additional file 4 — Figure S3. Relation between the number of variables and prediction accuracy. [file 1472-6807-10-20-S4.PDF]

Additional file 4

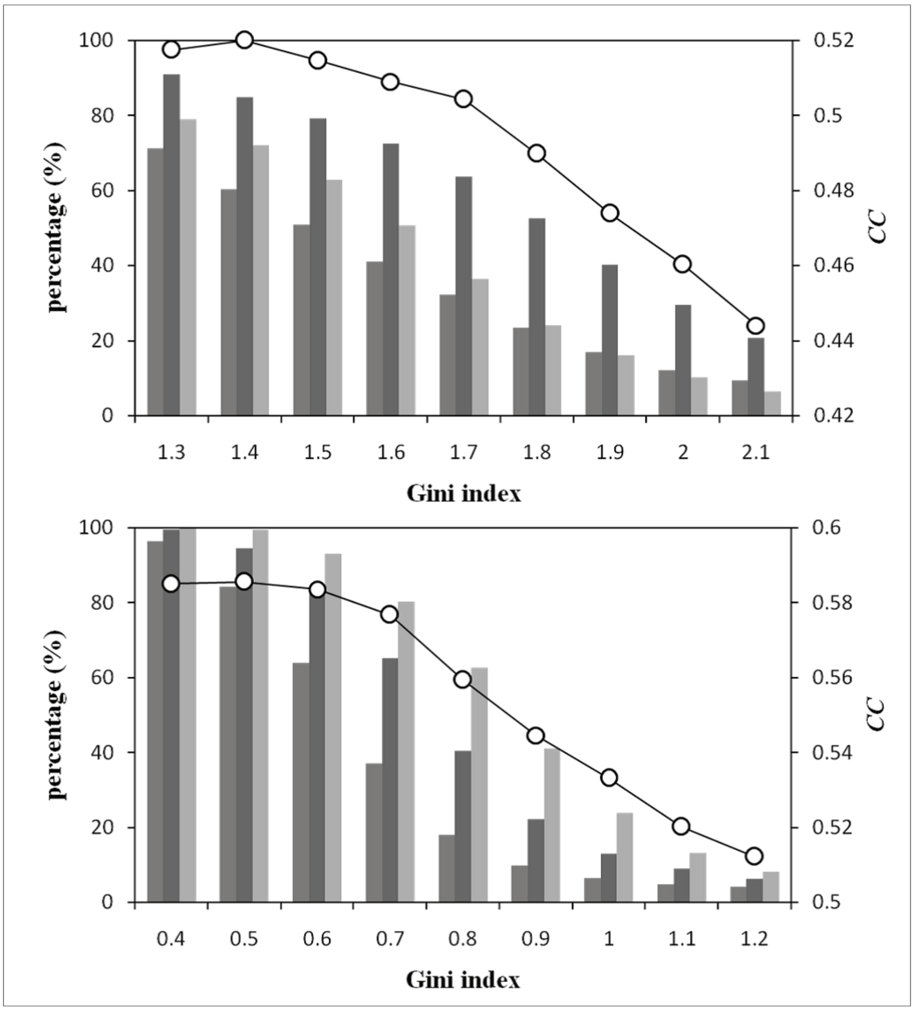

Figure S3 - Relation between the number of variables and prediction accuracy.

Plots of prediction accuracy in (A) internal motion, and (B) external_short motion are shown. The three histograms show the percentages of the numbers of variables included in the downsized model against the number of variables included in the original model. The three histograms for each Gini index value depict, from the left, models of CS, PS, and RS. The Gini index is shown on the horizontal axis. The percentages of the numbers of variables and the average of *CC*s are presented respectively on the left and right sides' vertical axes. These results were obtained using the method that implemented psipred and sable.
