# Supplementary material for: Prediction of protein motions from amino acid sequence and its application to protein-protein interaction
Source: BMC Struct Biol. 2010 Jul 13;10:20. doi: 10.1186/1472-6807-10-20 (PMC3245509; doi:10.1186/1472-6807-10-20)
Supplement: Additional file 5 — Figure S4. Proportion of features ranked in the top 20. [file 1472-6807-10-20-S5.PDF]

Additional file 5

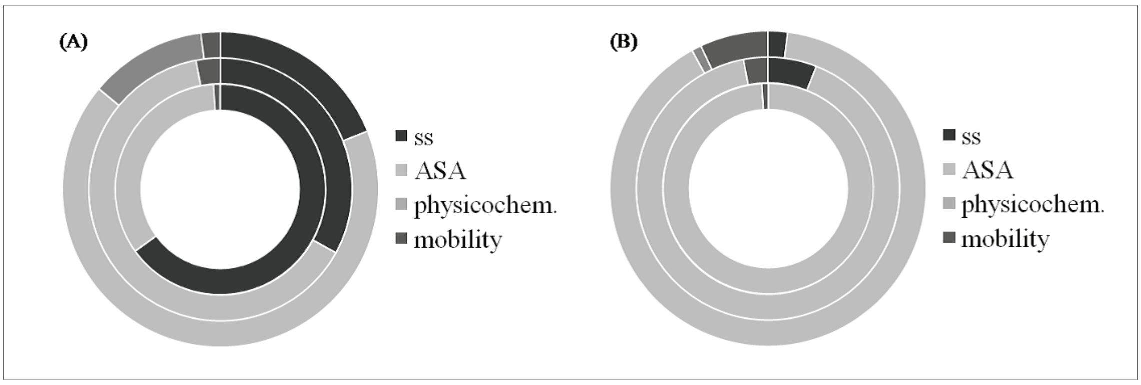

Figure S4 - Proportion of features ranked in the top 20.

Proportions of features in (A) internal motion, and (B) external_short motion are shown. The fractions represent shares of the variable category: ss, ASA, physicochem., and mobility. Definitions of these categories were presented in the Methods section. The circle charts signify, from the outside, the results of CS, PS, and RS models.
