# Supplementary material for: Prediction of protein motions from amino acid sequence and its application to protein-protein interaction
Source: BMC Struct Biol. 2010 Jul 13;10:20. doi: 10.1186/1472-6807-10-20 (PMC3245509; doi:10.1186/1472-6807-10-20)
Supplement: Additional file 6 — Figure S5. Prediction result of three published predictors for ection, Fab fragment, and erythropoietin. [file 1472-6807-10-20-S6.PDF]

Additional file 6

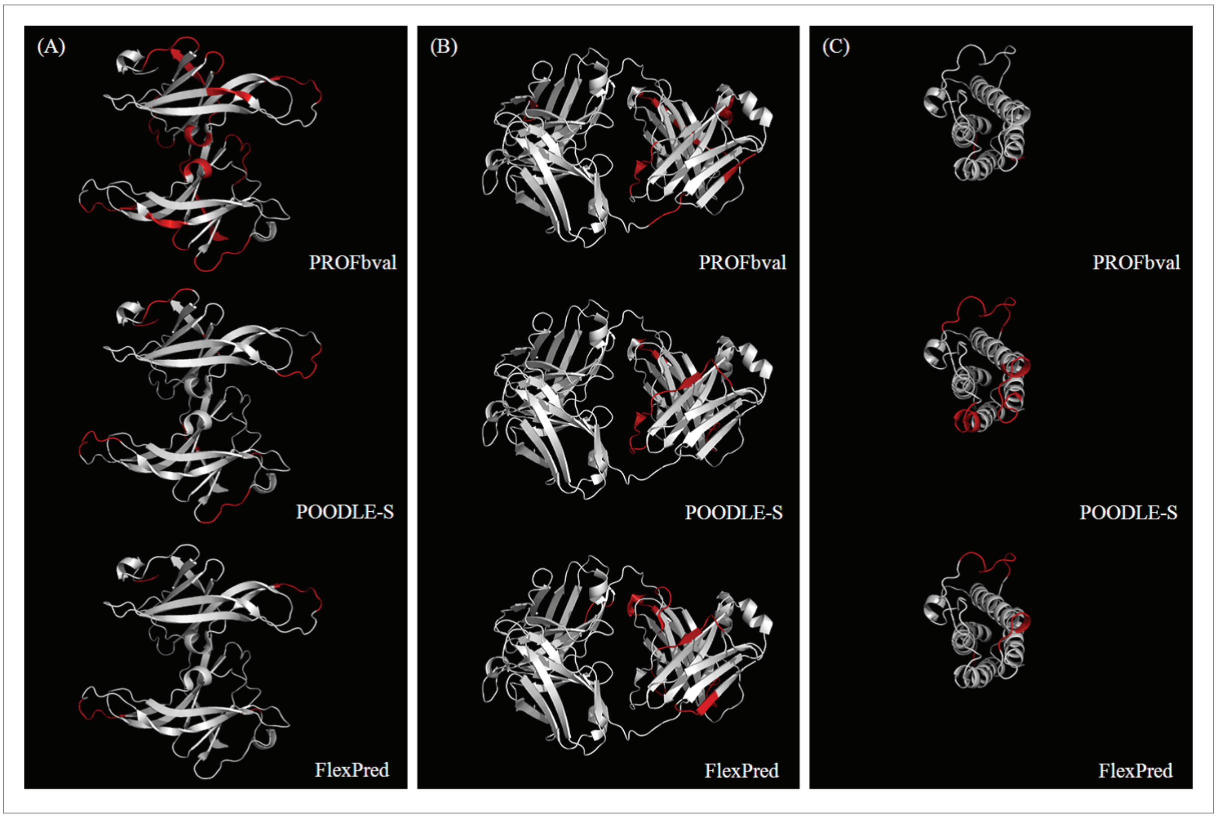

Figure S5 - Prediction result of three published predictors for (A) ecotin, (B) Fab fragment, and (C) erythropoietin.

The predicted high B-factor regions obtained from PROFbval, disordered regions obtained from POODLE-S and conformational switch regions obtained from FlexPred are mapped with red onto free-state structures. The definitions of candidate flexible regions obtained from three published predictors are the same as Table 3.
