# Supplementary material for: Prediction of protein motions from amino acid sequence and its application to protein-protein interaction
Source: BMC Struct Biol. 2010 Jul 13;10:20. doi: 10.1186/1472-6807-10-20 (PMC3245509; doi:10.1186/1472-6807-10-20)
Supplement: Additional file 7 — Table S2. List of prediction results for the large conformational change dataset. [file 1472-6807-10-20-S7.PDF]

Additional file 7

Table S2 - List of prediction results for the large conformational change dataset

Protein pair	PROFbval	POODLE-S	FlexPred	Proposed method
Staphylococcus A - Human Fc fragment	1–8, 21–26, 35–40<br>50–53, 55–60	1–12, 37–47, 51–60	1–5, 56–60	39–41
Ran GTPase - RCC1	1–4, 6–8<br>19–21<br>210–216	1–6, 128–136<br>175–202<br>208–216	1–7, 172–187<br>195–197<br>208–211	19–22, 32–34, 50–55<br>59–61, 129–132<br>170–189, 207–211
14-3-3 - Serotonin N-acetylate	69–73, 134–139,<br>207–212, 232–234<br>237–245	67–76, 139–143,<br>204–211<br>231–245	33–35, 67–73<br>110–112<br>230–245	31–38, 69–73<br>106–111, 134–136<br>229–233
Actin – Profili	1–5, 111–113<br>235–238, 241–243<br>324–327, 375–377	1–6<br>114–116<br>231–240	1–6, 232–237	22–24<br>109–116<br>230–236
Erythropoietin – EPO receptor	1–3, 163–166	23–31, 81–92<br>117–131	82–86, 119–128<br>164–166	26–30, 82–86<br>117–132
Fab fragment – Flu virus hemagglutinin	113–115, 125–128<br>161–171<br>207–210	158–174	159–165, 201–205	26–28, 57–60<br>107–115, 121–125<br>153–156, 200–205
	71–74<br>212–214<br>216–221	195–199, 211–214	7–10, 166–171<br>178–182, 194–199<br>209–213	7–17, 39–47<br>69–72, 119–123<br>132–134, 192–197
TGF-beta – TGF-beta receptor	1–3, 110–112	10–12, 68–72	2–13, 69–73	68–77, 95–101
Actin – Dnase I	1–5, 111–113<br>235–238, 241–243<br>324–327, 375–377	1–6<br>114–116<br>231–240	1–6, 232–237	22–24<br>109–116<br>230–236
Coagulation factor Vlla – Soluble tissue factor	1–4, 8–10<br>63–65, 163–167<br>183–191, 252–254	242–254	45–49, 62–67<br>137–139<br>241–247	74–81, 111–11<br>136–140, 164–167<br>188–191, 243–245
	1–5, 36–38, 58–60<br>96–99, 101–104	91–104	1–5, 88–91<br>102–104	75–88
Ran GTPase – Importin-beta	1–4, 6–8<br>19–21<br>210–216	1–6, 128–136<br>175–202<br>208–216	1–7, 172–187<br>195–197<br>208–211	19–22, 32–34, 50–55<br>59–61, 129–132<br>170–189, 207–211
HPr kinase C-ter domain – HPr	1–4, 72–75, 109–112<br>122–128, 133–137<br>192–205	1–9<br>129–131<br>192–205	123–128, 193–205	42–46<br>131–137<br>144–146
HIV reverse transcriptase – Fab28	18–20, 26–29<br>42–46, 66–70<br>99–105, 122–124<br>153–155, 172–174<br>215–224, 460–462<br>510–514<br>526–528<br>557–560	13–20<br>44–52<br>98–101<br>218–222<br>300–305<br>327–329<br>460–473<br>508–517	1–4<br>429–435<br>447–449	8–20, 44–54<br>92–95, 100–102<br>131–133, 141–145<br>152–157, 271–274<br>292–295, 333–336<br>390–393, 434–438<br>464–470, 488–492<br>509–512, 541–545
	18–20, 26–29<br>42–46, 66–70<br>99–104, 122–124<br>153–155, 171–175<br>215–224<br>248–251	13–21<br>44–53<br>98–101<br>219–222<br>300–306<br>327–329	1–4	8–21, 44–54<br>92–96, 99–107<br>131–133, 139–145<br>152–157, 214–216<br>271–274, 292–295<br>333–336, 390–393
Ecotin – D102N trypsin	14–19, 28–35<br>76–79, 89–94<br>102–104, 121–123<br>133–135, 137–142	1–3<br>14–18<br>83–89<br>135–147	1–3<br>88–93	12–20, 28–30<br>60–65<br>88–90<br>100–102
EPO receptor – Erythropoietin	1–4<br>10–12<br>37–39<br>210–213	56–63	2–4, 6–10, 34–38<br>51–56, 67–70<br>136–141, 154–159<br>165–168, 196–199	6–10, 38–49<br>67–70, 92–100<br>109–110, 133–138<br>150–165

Vitamin D binding – Actin	1–4, 112–114<br>133–138, 227–231<br>262–264, 274–280<br>305–307, 312–315<br>362–365, 411–418<br>455–458	192–194	60–64<br>114–116	36–41, 77–81<br>87–93, 175–178<br>226–229, 272–276<br>298–315, 319–323<br>413–417<br>436–439
Nitrogenase Fe – Nitrogenase Mo-Fe protein	1–3, 28–31, 51–53<br>63–71, 112–118<br>72–176, 190–192<br>221–224, 232–235<br>249–253, 260–262<br>274–279, 281–289	285–289	257–259	11–13<br>63–67<br>80–83<br>87–94<br>172–176<br>281–284
CDK2 – CDK inhibitor 3	9–11, 128–131<br>256–259<br>289–293	not detectable	135–139<br>204–207<br>243–240	20–22, 33–37<br>71–73, 95–98<br>153–156, 252–256
Gelsolin – Actin	4–7, 16–18<br>78–81, 89–92<br>115–118, 196–199<br>222–224<br>276–280<br>333–335	86–90<br>115–121<br>193–197<br>213–223	3–5, 44–46<br>114–123<br>222–225<br>296–300	6–9, 15–26, 79–84<br>86–90, 110–125<br>133–138, 146–151<br>169–173, 195–200<br>206–216, 244–248<br>296–299, 313–319
Importin-beta – Ran GTPase	65–69, 106–108<br>165–168, 182–188<br>210–212, 308–310<br>331–340, 375–377<br>397–401, 417–419<br>439–442	not detectable	48–50<br>140–142	10–14, 46–50<br>105–108<br>138–142<br>302–306<br>396–398<br>419–422
Hirustatin – Kallikrein	1–4,39–41, 52–55	1–17	1–5, 52–55	not detectable

Protein pairs and the definitions to determine candidates of conformational change region are identical to those shown in Table 3. "Not detectable" means that no candidate region exists. The regions that overlapped with observed conformational change regions are underlined.
