# Supplementary material for: Prediction of protein motions from amino acid sequence and its application to protein-protein interaction
Source: BMC Struct Biol. 2010 Jul 13;10:20. doi: 10.1186/1472-6807-10-20 (PMC3245509; doi:10.1186/1472-6807-10-20)
Supplement: Additional file 8 — Figure S6. Distribution of internal motion and observed conformational change for 20 proteins. [file 1472-6807-10-20-S8.PDF]

Additional file 8

Figure S6 - Distribution of internal motion and observed conformational change.

The red and blue solid lines respectively show the predicted scores for internal and external motion; the black dotted line signifies calculated scores from the observed conformational change upon protein–protein interaction. The residue number is shown on the horizontal axis. The locations of secondary structures are shown as red and yellow bars, respectively, for α-helix and β-sheet. The scores of two motions and observed conformational change are presented on the vertical axes. These results were obtained using the method that implemented psipred and sable.

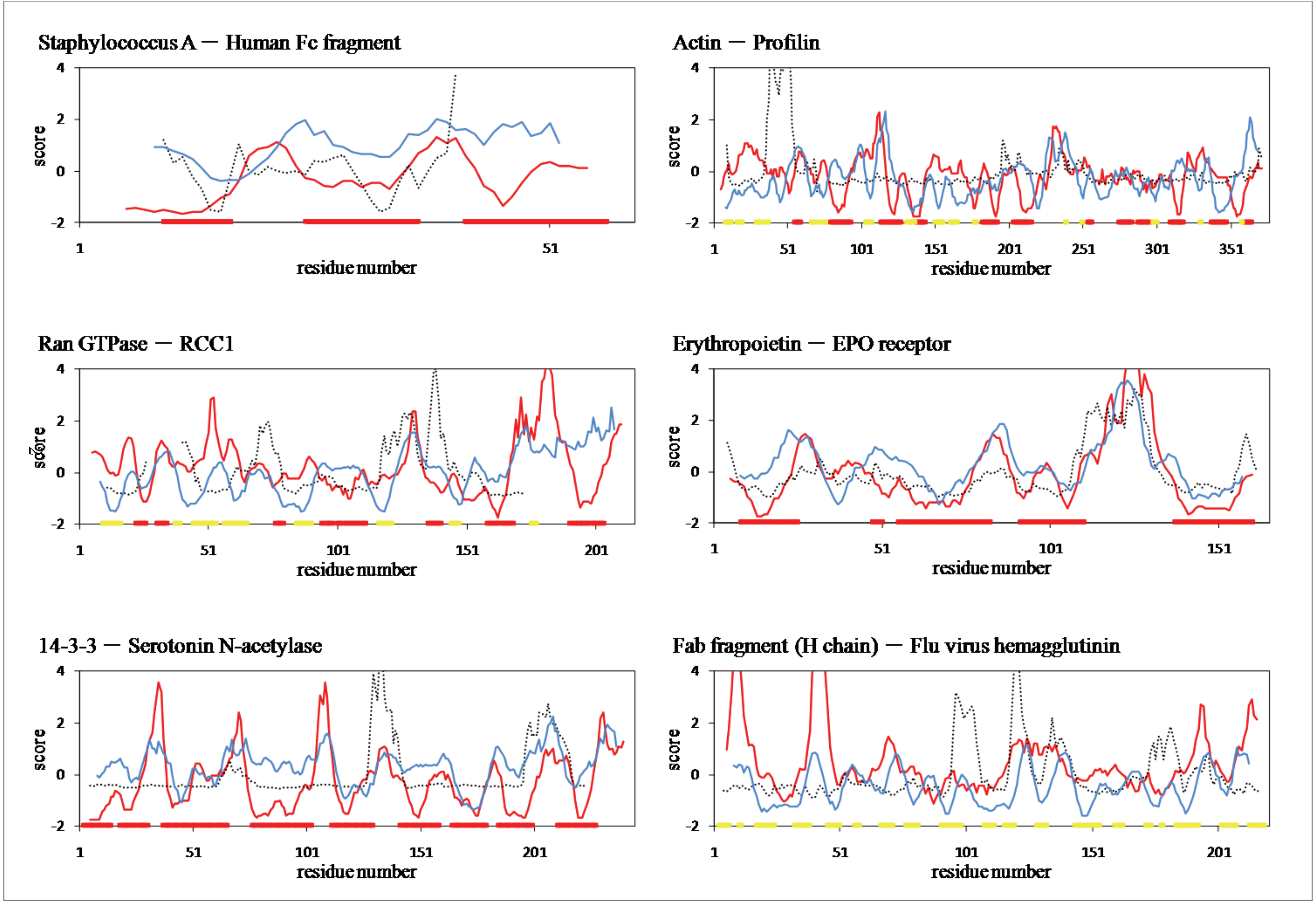
Staphylococcus A — Human Fc fragment
Actin — Profilin
Ran GTPase — RCC1
Erythropoietin — EPO receptor
14-3-3 — Serotonin N-acetylase
Fab fragment (H chain) — Flu virus hemagglutinin
score
residue number
4
2
0
-2
1
51
101
151
201
251
301
351

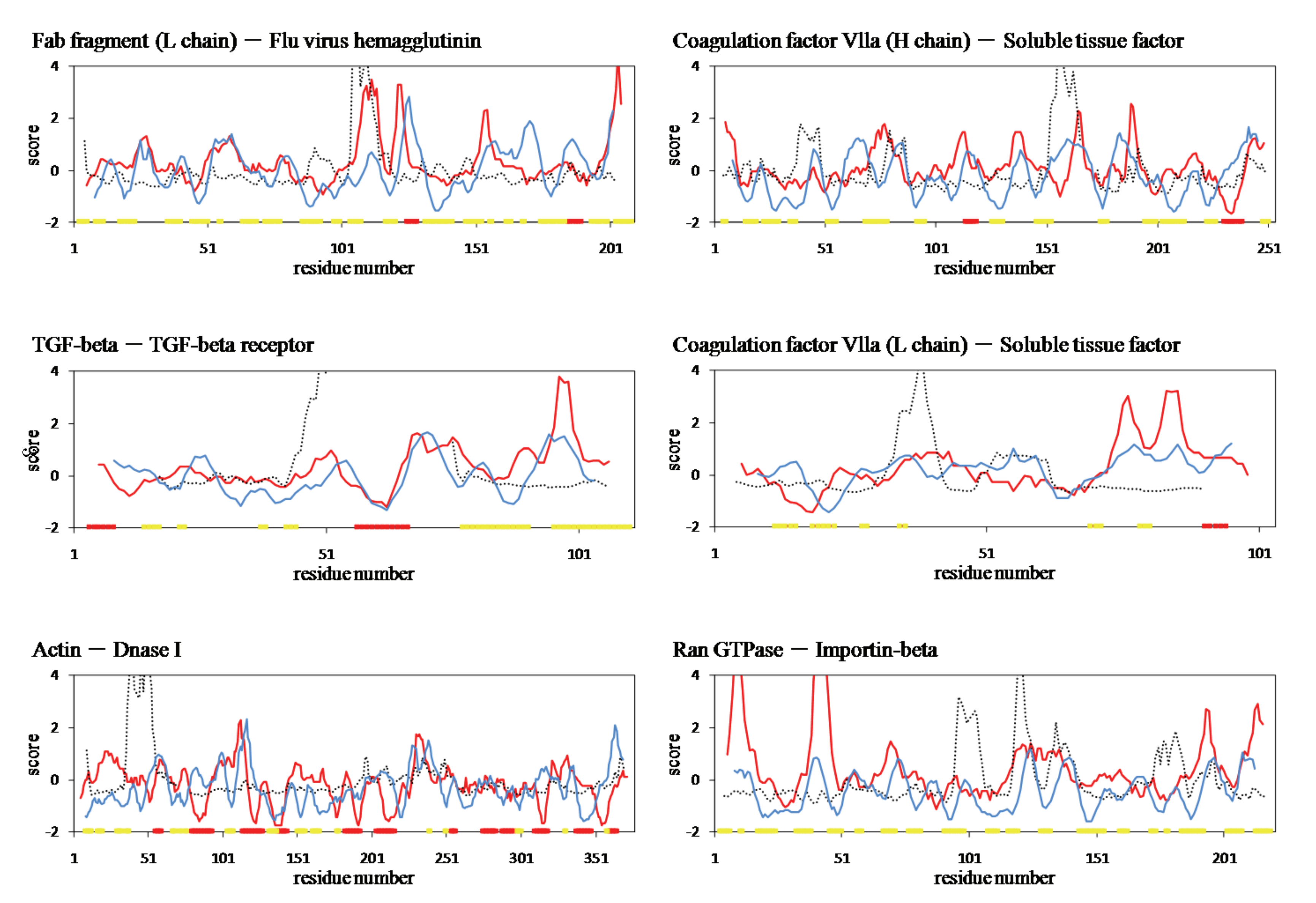
Fab fragment (L chain) — Flu virus hemagglutinin
Coagulation factor VIIa (H chain) — Soluble tissue factor
TGF-beta — TGF-beta receptor
Coagulation factor VIIa (L chain) — Soluble tissue factor
Actin — Dnase I
Ran GTPase — Importin-beta
score
residue number

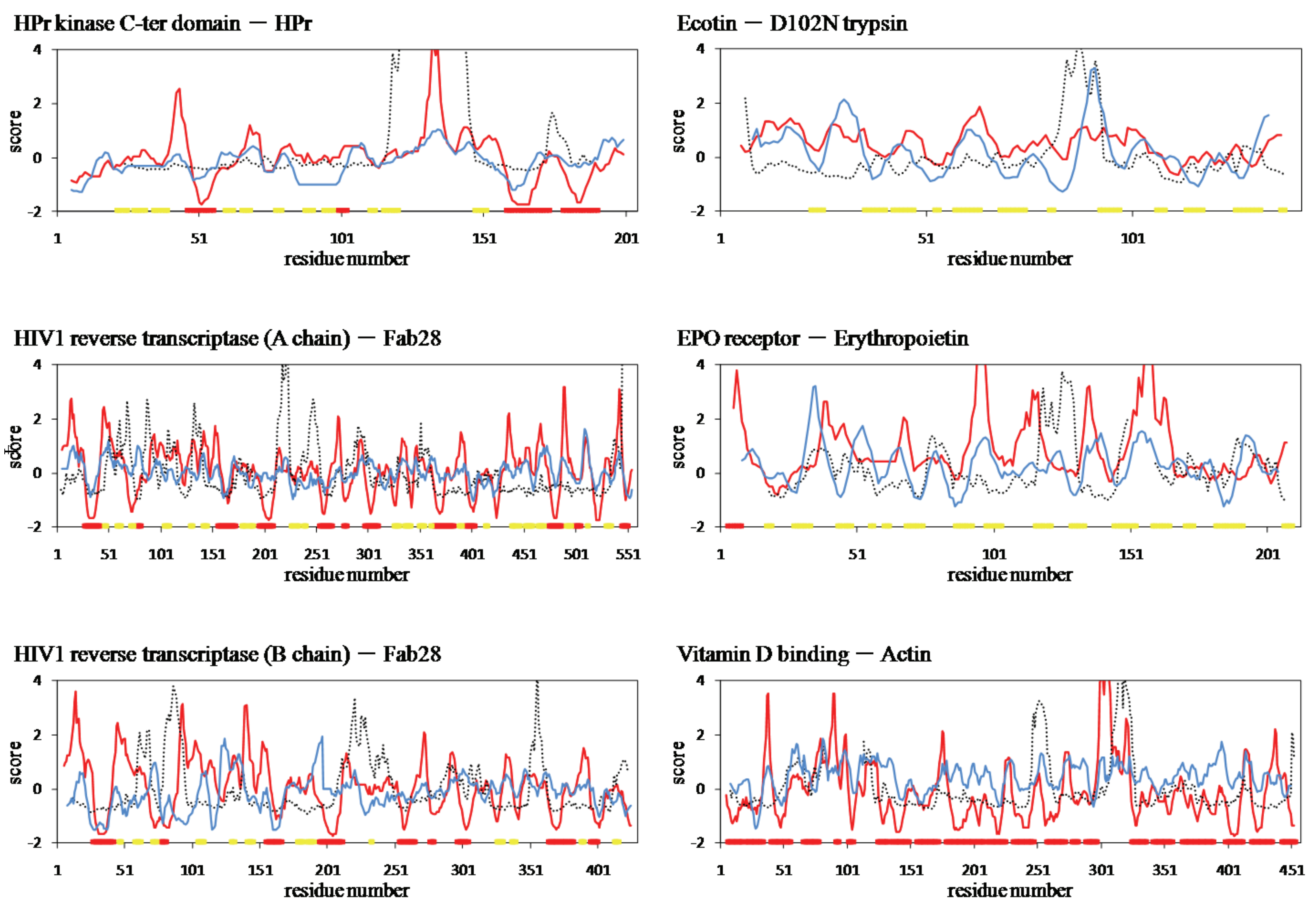

HPr kinase C-ter domain — HPr
Ecotin — D102N trypsin
HIV1 reverse transcriptase (A chain) — Fab28
EPO receptor — Erythropoietin
HIV1 reverse transcriptase (B chain) — Fab28
Vitamin D binding — Actin
score
residue number

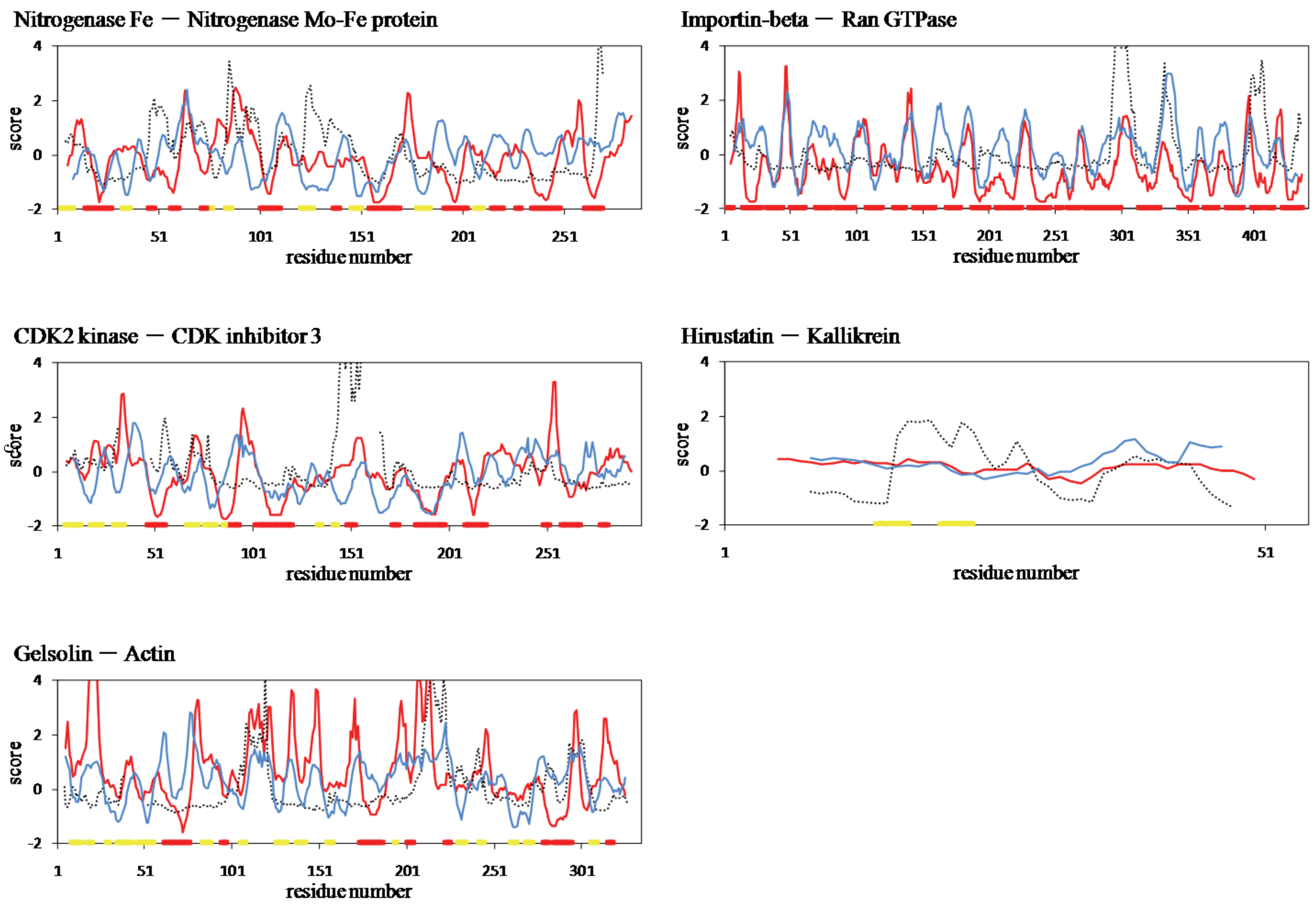
Nitrogenase Fe — Nitrogenase Mo-Fe protein
Importin-beta — Ran GTPase
CDK2 kinase — CDK inhibitor 3
Hirustatin — Kallikrein
Gelsolin — Actin
score
residue number
