# Supplementary material for: Prediction of protein motions from amino acid sequence and its application to protein-protein interaction
Source: BMC Struct Biol. 2010 Jul 13;10:20. doi: 10.1186/1472-6807-10-20 (PMC3245509; doi:10.1186/1472-6807-10-20)
Supplement: Additional file 9 — Figure S7. Influence of three parameters of random forest on the prediction error rate. [file 1472-6807-10-20-S9.PDF]

Additional file 9

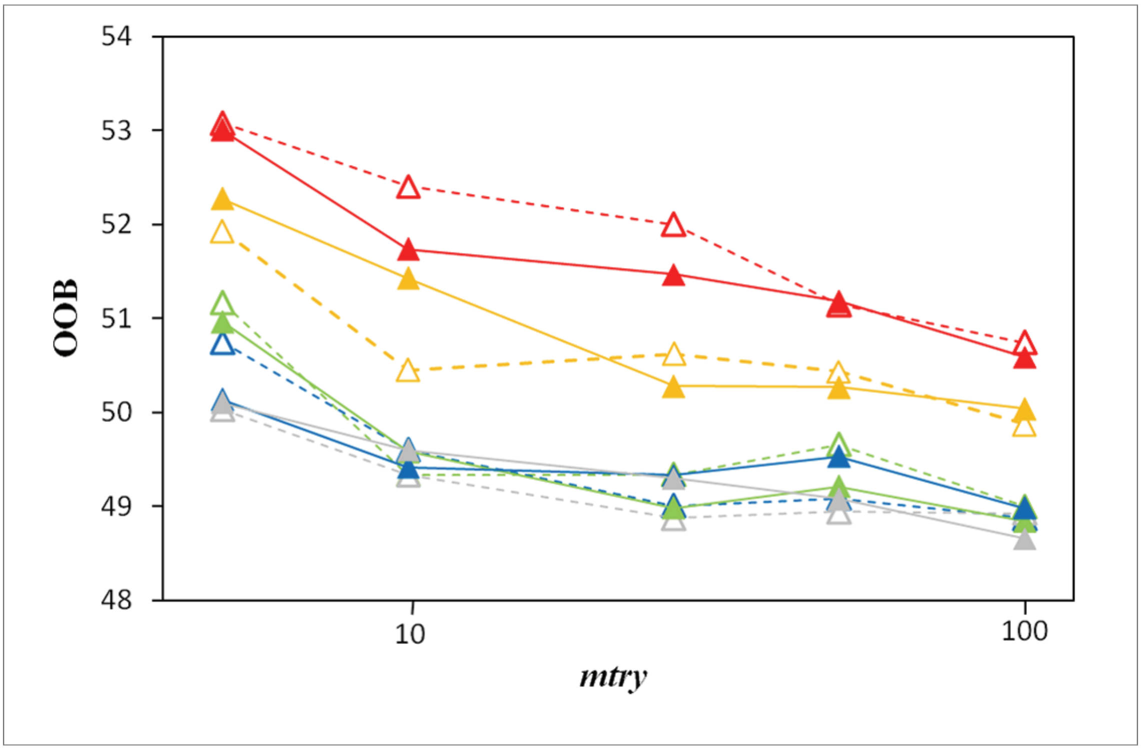

Figure S7 - Influences of three parameters of RF on the prediction error rate.

Results in CS of internal motion are shown for *mtry* = {5, 10, 27, 50, 100}, *ntree* = {100, 200, 500, 1000, 2000}, and *nodesize* = {1, 5}. The Out-of-Bag (OOB) and *mtry* are shown respectively on the vertical and horizontal axes. The OOB is an indicator used to estimate the classification error. The horizontal axis scale is logarithmic. Red, orange, green, blue, and gray respectively denote 100, 200, 500, 1000, and 2000 in *ntree*. The bold and dotted lines signify 1 and 5 in *nodesize*. The default values of *mtry*, *ntree*, and *nodesize* are, respectively, 27, 500, and 1.
