# Supplementary material for: Prediction of protein motions from amino acid sequence and its application to protein-protein interaction
Source: BMC Struct Biol. 2010 Jul 13;10:20. doi: 10.1186/1472-6807-10-20 (PMC3245509; doi:10.1186/1472-6807-10-20)
Supplement: Additional file 10 — Table S3. The average and standard deviation of normalized NMA score in each category. [file 1472-6807-10-20-S10.PDF]

Additional file 10

Table S3 – The average and standard deviation of normalized NMA score in each category

Motion	Flexible		Normal		Rigid	
	avg.	*SD*	*avg.*	*SD*	*avg.*	*SD*
internal motion	-0.248	0.919	0.138	0.969	0.423	1.067
external_short	-0.309	0.783	-0.056	0.841	0.179	0.908
external_long	-0.301	0.813	-0.076	0.883	0.188	0.990

Herein, *avg.* and *SD* respectively signify the average and standard deviation of the normalized NMA score in each category.
